# Prediction of Novel Inhibitors of the Main Protease (M-pro) of SARS-CoV-2 through Consensus Docking and Drug Reposition

**DOI:** 10.3390/ijms21113793

**Published:** 2020-05-27

**Authors:** Aleix Gimeno, Júlia Mestres-Truyol, María José Ojeda-Montes, Guillem Macip, Bryan Saldivar-Espinoza, Adrià Cereto-Massagué, Gerard Pujadas, Santiago Garcia-Vallvé

**Affiliations:** 1 Departament de Bioquímica i Biotecnologia, Research group in Cheminformatics & Nutrition, Campus de Sescelades, Universitat Rovira i Virgili, 43007 Tarragona, Catalonia, Spain; aleix.givi2@gmail.com (A.G.); julia.mestres@estudiants.urv.cat (J.M.-T.); guillem.macip@gmail.com (G.M.); bsaldivar.emc2@gmail.com (B.S.-E.); ssorgatem@gmail.com (A.C.-M.); 2 Escoles Universitàries Gimbernat i Tomàs Cerdà, 08174 Sant Cugat del Vallès, Barcelona, Catalonia, Spain; mjoseom88@gmail.com; 3 EURECAT, TECNIO, CEICS, Avinguda Universitat 1, 43204 Reus Catalonia, Spain

**Keywords:** SARS coronavirus, COVID-19, SARS-CoV-2, 3CL-pro, M-pro, chymotrypsin-like protease, 2019-nCov

## Abstract

Since the outbreak of the COVID-19 pandemic in December 2019 and its rapid spread worldwide, the scientific community has been under pressure to react and make progress in the development of an effective treatment against the virus responsible for the disease. Here, we implement an original virtual screening (VS) protocol for repositioning approved drugs in order to predict which of them could inhibit the main protease of the virus (M-pro), a key target for antiviral drugs given its essential role in the virus’ replication. Two different libraries of approved drugs were docked against the structure of M-pro using Glide, FRED and AutoDock Vina, and only the equivalent high affinity binding modes predicted simultaneously by the three docking programs were considered to correspond to bioactive poses. In this way, we took advantage of the three sampling algorithms to generate hypothetic binding modes without relying on a single scoring function to rank the results. Seven possible SARS-CoV-2 M-pro inhibitors were predicted using this approach: Perampanel, Carprofen, Celecoxib, Alprazolam, Trovafloxacin, Sarafloxacin and ethyl biscoumacetate. Carprofen and Celecoxib have been selected by the COVID Moonshot initiative for in vitro testing; they show 3.97 and 11.90% M-pro inhibition at 50 µM, respectively.

## 1. Introduction

The recently worldwide pandemic named COVID-19 (COronaVIrus Disease 2019) has spread rapidly since it emerged in Wuhan (China) in December 2019. SARS-CoV-2 has been identified as the pathogen responsible for the outbreak of an atypical pneumonia whose symptoms range from mild effects such as fever, dry cough, fatigue, dyspnea, difficulty breathing, to severe progressive pneumonia, multiorgan failure and death [1]. Since the outbreak of SARS-CoV-2, the World Health Organization (WHO) has declared a state of global health emergency. Thus, as of the 15th of May 2020, the total number of confirmed cases of COVID-19 has risen to 4,434,590 in at least 188 different countries. Likewise, more than 301,937 deaths and 1,583,929 cases of recovery have been reported according to the Johns Hopkins Coronavirus map tracker [2] at https://coronavirus.jhu.edu/map.html. The risk of severe cases increases in elderly patients with previous pathologies, such as heart failure or diabetes (i.e., 89.5% of deaths in Italy for COVID-19 have been among people over 70 years old) [3].

The pathogen that caused the pandemic has been identified as a novel coronavirus which belongs to the *β-coronavirus* family; it is related to acute respiratory syndrome coronavirus (SARS-CoV), which caused another outbreak in 2003 [4,5]. Currently, there are no targeted therapeutics or effective treatments against this new virus. Because of that, the scientific community is making great efforts to investigate different mechanisms to interfere with the virus’ metabolism. As a consequence, several antiviral drugs used in patients with similar viral infections have been tested in recent clinical trials against COVID-19, including Remdesivir (designed for the Ebola virus [6]), Lopinavir/Ritonavir (designed for the HIV [1]), chloroquine and hydroxychloroquine (designed for malaria [6]) and Tocilizumab (designed for rheumatoid arthritis [7]), among others. Nevertheless, the efficacy of some drugs remains controversial. This is the case with a clinical trial involving Lopinavir/Ritonavir, which reported that no benefits were observed with this treatment compared to standard care [1].

The characterization of main protease (M-pro), also known as chymotrypsin-like protease (3CL-pro), has emerged as one of the key targets for the development of antiviral therapies aimed at blocking the life cycle of the coronavirus [4,8,9,10]. M-pro is found in the polyprotein ORF1ab of SARS-CoV-2 and is essential for the replication of the virus. This protease is involved in the cleavage of polyproteins, a process that produces nonstructural proteins that are part of the replicase-transcriptase complex [8,10]. The sequence of the M-pro enzyme has a high identity (i.e., >96%) with SARS-CoV, except for a key residue (i.e., Ala285Thr), which may contribute to the high infectivity of the virus [11]. Moreover, a high superposition correlation (with a value of 0.44 Å for C^α^ RMSD) has been found between the M-pro structure SARS-CoV (i.e., PDBid 3D62) and the recently crystallized structure of SARS-CoV-2 (i.e., PDBid 6LU7) [9,12]. Therefore, besides the fact that this enzyme only exists in the virus and not in humans [8], the high conservation of M-pro among the related viruses and its importance in the replication of the virus makes this enzyme an attractive target for potential antiviral drugs [12]. As a result, the structure of M-pro has been recently solved in different conditions by X-ray crystallography.

Computational approaches can make a great contribution to drug discovery by reducing cost and time (especially for emerging diseases such as COVID-19) and speeding up analyses of target interactions with drug candidates [13]. Consequently, different computational studies have been published in order to better understand the mechanism of M-pro and try to inhibit its function [4,5,10,12,14,15,16,17,18,19,20,21]. Nevertheless, it is important to highlight the fact that, despite the high level of similarity of SARS-CoV-2 with other members of the coronavirus family, their binding sites have differences in shape and size which mean that repurposing SARS drugs may not be successful, and enhanced sampling should be considered [1,19]. Consequently, although the development of a more specific inhibitor is highly desirable, in the absence of an effective treatment, drug repurposing becomes an attractive solution, because it reduces the time and cost of drug development [22]. This strategy is a promising way to explore alternative indications and to identify new targets for existing drugs which have already been established as safe. As the safety profiles of these drugs have already been demonstrated, clinical trials for alternative indications are cheaper, potentially faster and carry less risk than traditional drug development [22].

Therefore, the main goal of this study is to apply an original virtual screening (VS) protocol in order to identify high affinity docked poses that are simultaneously predicted by three different docking programs. This allows us to rapidly identify commercial drugs that have high potential to inhibit M-pro, and subsequently, be tested as a treatments against COVID-19.

## 2. Results and Discussion

### 2.1. Structural Description of M-Pro and Report of Known Mutations in Its Structure

**Figure 1:**
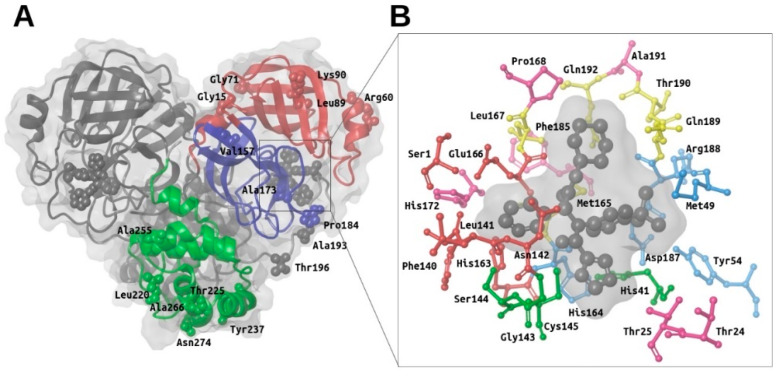
Overview of the main structural features of the SARS-CoV-2 M-pro. Panel (**A**) shows a general overview of the M-pro homodimeric structure (PDBid 6W63) and the relative location of the missense mutations shown in Table 1. The domain structure for one of the two protomers is also shown (with domain I in red, domain II in blue and domain III in green). Panel (**B**) shows the most important residues from the different subsites in the context of the binding site. Thus, the residues from the S_3_, S_2_, S_1_ and S_1_’ subsites are shown in yellow, cyan, red and green, respectively. Other important residues at the binding site that have not been assigned by Tang et al. to any of these subsites are shown in magenta. This figure was obtained with the help of the Maestro program [24].

SARS-CoV-2 M-pro is a homodimeric protein with two subunits related by a crystallographic 2-fold symmetry axis (see Figure 1A) [9]. Each subunit (also called *protomer*) has a length of 306 residues and is formed by three domains (i.e., domain I from residues 8 to 100, domain II from residues 101 to 184 and domain III from residues 199 to 306). Domains I and II share the same fold (an antiparallel six-stranded β-barrel structure), whereas domain III is formed by five α-helices arranged into a largely antiparallel globular cluster. Domains II and III are connected by a long loop formed by residues from 185 to 198. The substrate-binding site of M-pro is located at a cleft between domains I and II, whereas domain III is involved in regulating M-pro dimerization through an intersubunit salt-bridge between Glu290 (from one protomer) and Arg4 (from the other protomer) [8]. The formation of this dimer is essential for M-pro activity because the N-terminal residue of one protomer (i.e., Ser1) interacts with the Glu166 of the other, and thus, helps to form the S_1_ subsite of the substrate-binding pocket [8].

The enzyme has a catalytic dyad formed by His41 and Cys145. As in any protease, other important subsites at the M-pro binding site are S_3_, S_2_, S_1_ and S_1_′, that are occupied, respectively, by the P_3_, P_2_, P_1_ and P_1_′ residues of its peptidic substrate (where the point of peptide cleavage is at the peptide bond that binds residue P_1_ with residue P_1_′). Thus, according to Tang et al. [12], S_3_ is formed by Met165, Leu167, Gln189, Thr190 and Gln192 (see yellow residues in Figure 1B); S_2_ is formed by Met49, Tyr54, His164, Asp187 and Arg188 (see cyan residues in Figure 1B); S_1_ is formed by Ser1 (from the other protomer), Phe140, Leu141, Asn142, His163 and Glu166 (see red residues in Figure 1B); and S_1_’ is formed by His41, Gly143, Ser144 and Cys145 (see green residues in Figure 1B) [12]. Other important residues identified by different authors at the M-pro binding site are Thr24, Thr25, Pro168, His172, Phe185 and Ala191 (see magenta residues in Figure 1B) [8,9].

According to the data obtained from GISAID [23], 16 missense mutations have been identified to date in the SARS-CoV-2 gene that codes for M-pro (see Table 1). For the moment, these mutations do not affect residues at the binding site, although some of them (i.e., Ala173, Pro184 and Ala193) occur at its proximity (see Figure 1A).

**Figure 2:**
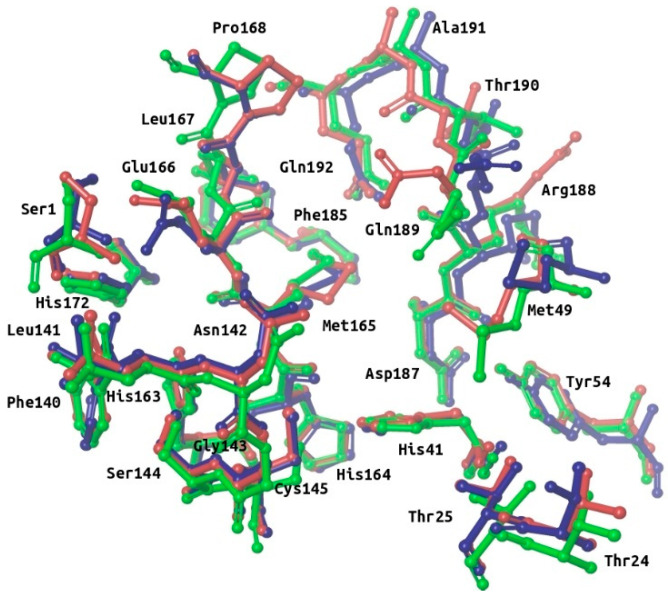
Superposition of the binding site residues of SARS-CoV-2 M-pro. Residues corresponding to the free enzyme (PDBid 6M03), M-pro bound to a noncovalent inhibitor (PDBid 6W63) and M-pro bound to a covalent inhibitor (PDBid 6LU7) are shown in red, blue and green, respectively. This figure was obtained with the help of the Maestro program [24].

Many M-pro structures have been recently deposited at the PDB (see Table S1). The first M-pro structure that was delivered by the PDB was 6LU7 [9] and, therefore, this has been the PDB file of choice for the different VS papers or structure-based drug designs reported that do not use homology models [5,12,21,25,26]. Figure 2 shows that when 6LU7 is superposed to 6W63 (M-pro bound to a noncovalent inhibitor) or to 6M03 (free enzyme), only minor structural changes affect the binding site structure: (a) Met49/Arg188 (S_2_ subsite) and Met165/Gln189 (S_3_ subsite) show totally different conformations of their sidechains (while keeping Met49 and Arg188 main chains coincident); (b) all the residues at the S_3_ subsite, Pro168 and Ala191 have their main chain slightly displaced in one or in all three structures; and (c) Ser1 and Asn142 (S_1_ subsite) show a slight change affecting the end of their side chains. When the coordinates of all of these residues are compared to their corresponding electron density map (EDM), all of them are correctly modeled, except for Gln189 from 6M03/6W63 and Met165 from 6M03. Then, given that the modeling of the binding site residues relative to the EDM is better in 6LU7 than in 6M03 or 6W63, we performed all our protein-ligand docking runs with 6LU7 as the target structure. Moreover, considering that: (1) our structural analysis has shown that the M-pro binding site has very limited flexibility; (2) the most flexible residues are Met49/Arg188 (S_2_ subsite) and Met165/Gln189 (S_3_ subsite); (3) the S_1_’ pocket is rigid; and (4) in the S_1_ subsite changes only slightly at the end of the Ser1 and Asn142 side chains; we have decided that the receptor binding site will be considered as rigid in all the protein-ligand docking runs performed in this work, and that it will only be considered to be flexible when performing a Molecular Mechanics Generalized Born model and Solvent Accessibility (MM-GBSA) calculation to minimize the hit poses that succeed in the VS workflow.

### 2.2. Description of the Intermolecular Interactions between M-Pro and Cocrystallized or Predicted Inhibitors

**Figure 3:**
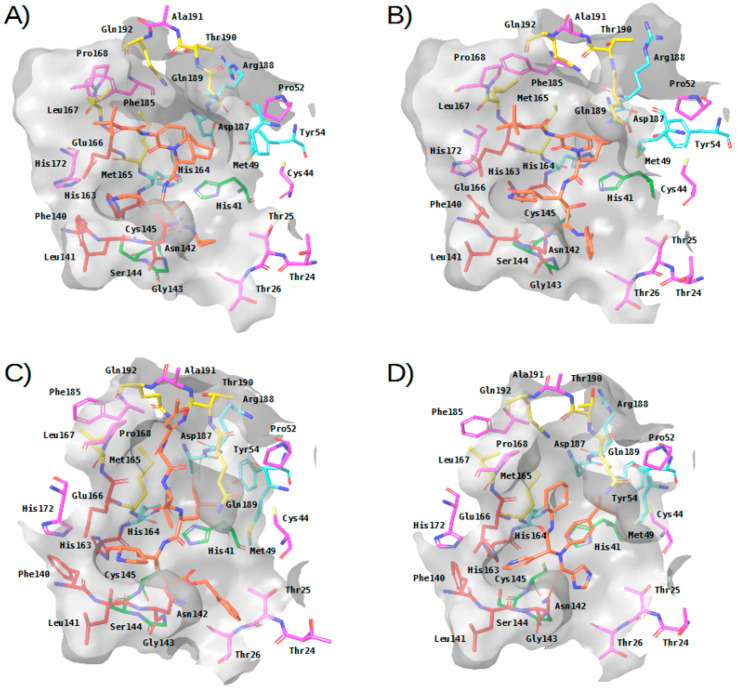
Three-dimensional view of the binding site in experimental complexes between M-pro and reversible and irreversible inhibitors. Panels (**A**–**D**) correspond to complexes with **13a** (PDBid 6Y7M), **13b** (PDBid 6Y2F), **N3** (PDBid 6LU7) and **X77** (PDBid 6W63), respectively. This figure was obtained with the help of the Maestro program [24].

Until now, four M-pro inhibitors have been cocrystallized with M-pro [8,9,27]. Three of these cocrystallized ligands (i.e., **13a**, **13b** and **N3**) are irreversible inhibitors that bind to M-pro through a covalent bond with Cys145, while the fourth (i.e., **X77**) is a reversible inhibitor. Table 2 shows the intermolecular interactions between M-pro and these four ligands. Table 3 shows a summary of the relevance of each binding site residue in the intermolecular interactions between M-pro and the docked poses of the top 30% compounds with the highest M-pro predicted affinity from four reference libraries [a general anti-SARS library (i.e., OTAVA-ML-SARS) and three libraries containing predicted M-pro inhibitors (i.e., OTAVA-SARS-CoV-2, COVID-Moonshot and DD-top-1000 [5]]. A comparison of Table 2 and Table 3 reveals that the following interactions are common between cocrystallized ligands and the docked poses obtained for the compounds in the reference libraries and, therefore, may constitute important interactions:

S_3_ and S_2_ subsites:
  - In the four experimental complexes, Met165 and Gln189 pin the ligand from both sides at the S_3_ subsite through hydrophobic interactions, with Gln189 interacting with two of its side chain carbons (i.e., CG and CB) and Met165 interacting with two of its side chain atoms (i.e., CB and SD; see Table 2 and Figure 3). Most of the compounds in the reference libraries also interact with Met165 and Gln189 (see Table 3). His41, Met49 and Asp187 also present hydrophobic interactions with most of the ligands around this area (see Table 2 and Figure 3). Met49 interacts with the ligands via its side chain atoms (i.e., CB, CG, SD and CE), whereas Asp187 and His41 use their CB carbon atom. Table 3 also shows that His41 and Met49 are highly important in the intermolecular interactions with the compounds from the reference libraries (with a more modest role for Asp187). Therefore, all these hydrophobic interactions would act as a hydrophobic grip around the same ligand group and greatly contribute to its binding affinity, which would explain the presence of the highly hydrophobic groups that the cocrystallized ligands present in this position (i.e., cyclohexylmethyl for **13a**, cyclopropylmethyl for **13b**, isopropylmethyl for **N3** and t-butyl for **X77**; see Figure 3).
  - The carbonyl oxygen of His164 (a residue close to the previously described hydrophobic region) provides an anchor point for **13b**, **N3** and **X77** by acting as a hydrogen bond acceptor (see Table 2 and Figure 3). The interaction with His164 also seems important for a high percentage of docked poses in the COVID-Moonshot and DD-top-1000 reference libraries (see Table 3).
S_1_ subsite:
  - In the S_1_ subsite, the carboxylic acid group of Glu166 is able to establish either a hydrogen bond interaction with **13b** or a salt bridge with **N3**. Moreover, its main chain oxygen and nitrogen (both oriented towards the S_3_ subsite) are able to act respectively as a hydrogen bond acceptor with ligands **13a**, **13b** and **N3** or as a hydrogen bond donor with all the ligands (see Table 2 and Figure 3). Therefore, the high number of interactions between this residue and different parts of the ligand suggest that it plays a key role in the binding of compounds. In fact, at least half of the compounds in the four reference libraries interact with this residue (see Table 3).
  - The oxygen main chain of Phe140 is able to establish a hydrogen bond with ligands **13a**, **13b** and **N3** (see Table 2 and Figure 3), but few docked poses in the reference libraries interact with this residue (see Table 3).
  - The side chain of His163 is able to effect hydrogen bond interactions with **13a** and **X77** (see Table 2 and Figure 3), but few docked poses in the reference libraries interact with this residue (see Table 3).
S_1_′ subsite:
  - In the S_1_′ subsite, **13a**, **13b** and **N3** bind covalently to the catalytic residue Cys145, and **13b** and **N3** effect a hydrogen bond interaction with the NE2 atom of His41 (see Table 2 and Figure 3). As Cys145 and His41 constitute the catalytic dyad of M-pro, interacting with these residues may be key to establishing a strong binding with this enzyme. Although few of the docked poses of the compounds in the four reference libraries interact with Cys145, most of them interact with His41 (see Table 3).
  - In addition, the main chain nitrogen atom of Gly143 effects hydrogen bond interactions with all the cocrystallized ligands, and many compounds in the reference libraries also interact with this residue (see Table 2 and Table 3 and Figure 3). Interacting with Gly143 may be important to orient the compound towards the S_1_’ subsite and stabilize the binding of the compound in the catalytic site.

Overall, three main regions can be highlighted in the M-pro binding site based on the analysis of crystallized protein-ligand complexes: (1) a hydrophobic pocket formed by the S_3_ and S_2_ subsites, in which a set of hydrophobic interactions by the residues Gln189, Met165, Met49, Asp187 and His41 (together with a hydrogen bond interaction with the main chain carbonyl oxygen of His164), contribute to a hydrophobic grip of the ligand; (2) the S_1_ pocket, in which Glu166 uses its main chain nitrogen and oxygen atoms to effect hydrogen bond interactions with all the cocrystallized ligands; and (3) the S_1_’ subsite, to which the ligand is fixed by covalent and noncovalent interactions with the residues Cys145 and His41 of the catalytic dyad and a hydrogen bond interaction with the Gly143 main chain nitrogen.

### 2.3. Virtual Screening of Approved Drugs

**Figure 4:**
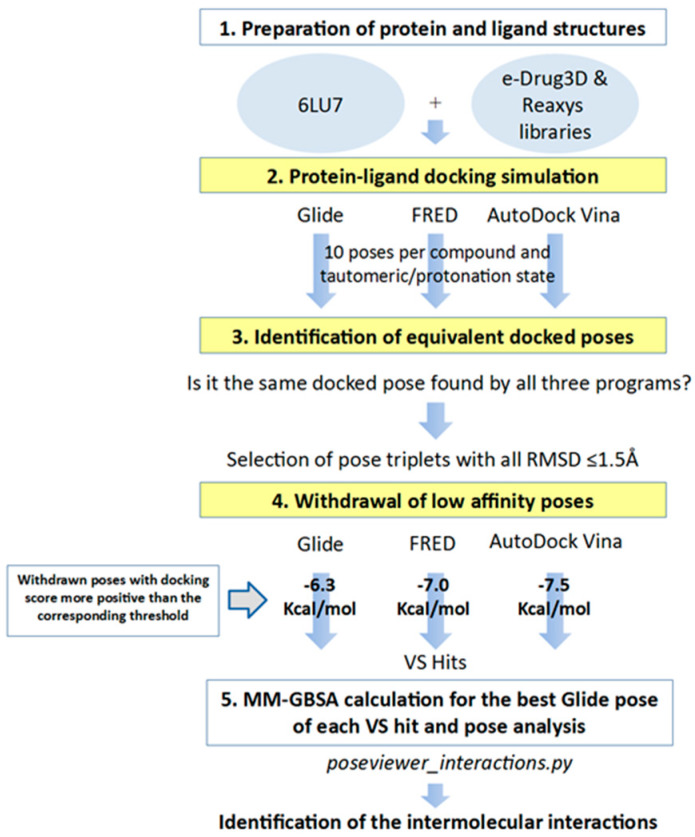
General scheme of the workflow used for approved-drug reposition. VS screening steps are shown against a yellow background to distinguish them from preparation or results postprocessing steps.

In order to identify inhibitors that could bind to the binding site of M-pro, in the VS developed herein, we analyzed the results of three different protein-ligand programs (i.e., Glide, FRED and AutoDock Vina) and compared them to obtain the docked poses that could be obtained simultaneously with all of them. Glide, FRED and AutoDock Vina follow different approaches to generate docked poses and score the results. Glide and FRED use different exhaustive algorithms to obtain docked poses, while AutoDock Vina uses an iterated local search global optimizer [28,29]. Regarding their scoring functions, while the Glide and FRED scoring functions are fully empirical [30,31], the scoring function of AutoDock Vina is a hybrid scoring function that incorporates empirical and knowledge-based elements [32]. Because of the differences between these three protein-ligand docking programs, both in their search algorithms and scoring functions, focusing on their intersection should compensate for their individual weaknesses [33,34]. With this in mind, we designed a VS (see Figure 4) that consisted of the following three steps: (1) performing independent protein-ligand docking simulations with Glide, FRED and AutoDock Vina; (2) identifying equivalent docked poses among the three docking programs (referred to as triplets for simplicity); and (3) applying a docking score threshold to consider as VS hits only the equivalent docked poses with high affinity for M-pro. Then, if at least one of the three equivalent poses that form a triplet has a higher positive docking score than the corresponding threshold value, then this triplet is removed from the VS hits list. Also, if more than one triplet was found for the same hit, the one that presented the highest mean docking score was chosen. Finally, the Glide pose of each VS hit triplet was submitted to an energy minimization with the binding site of M-pro by using the MM-GBSA minimization available at Prime [35].

**Figure 5:**
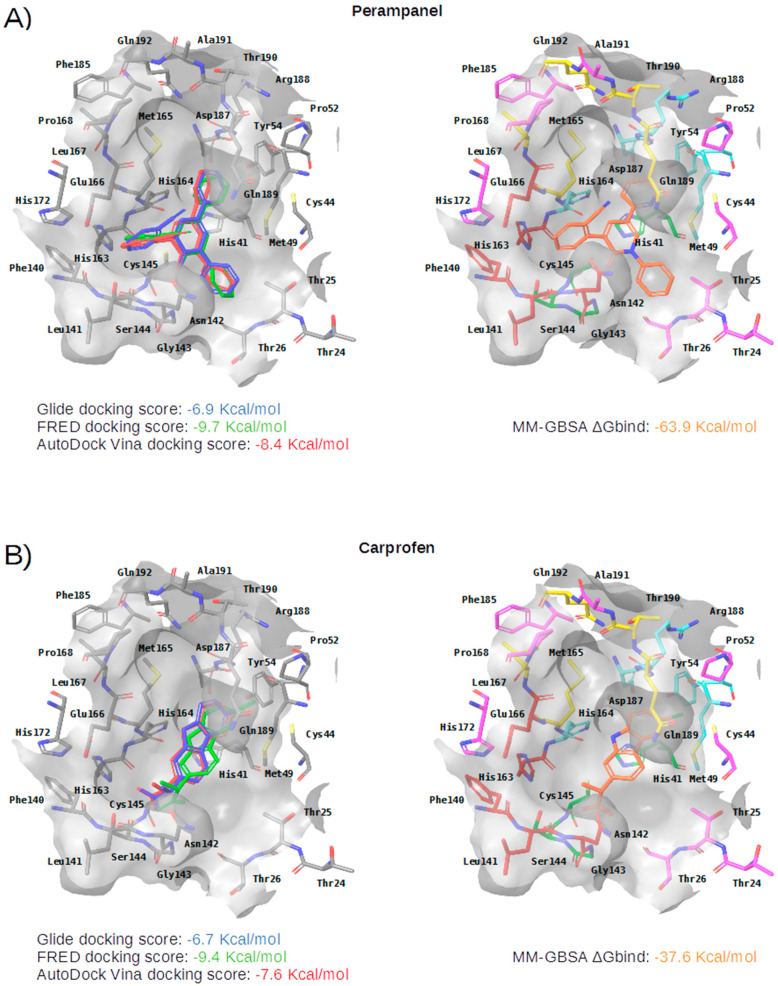

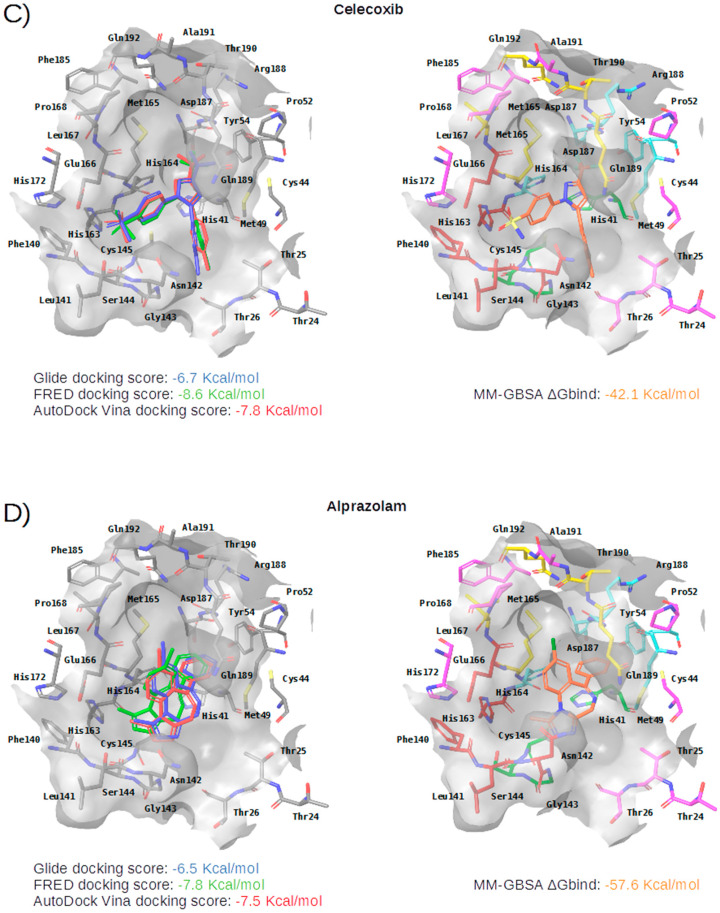

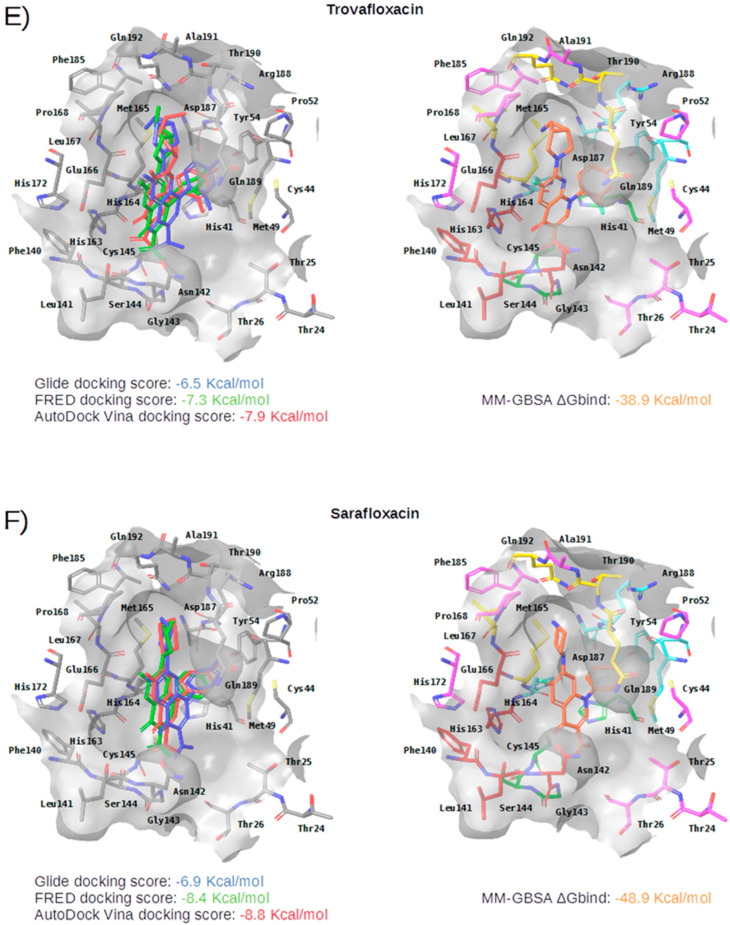

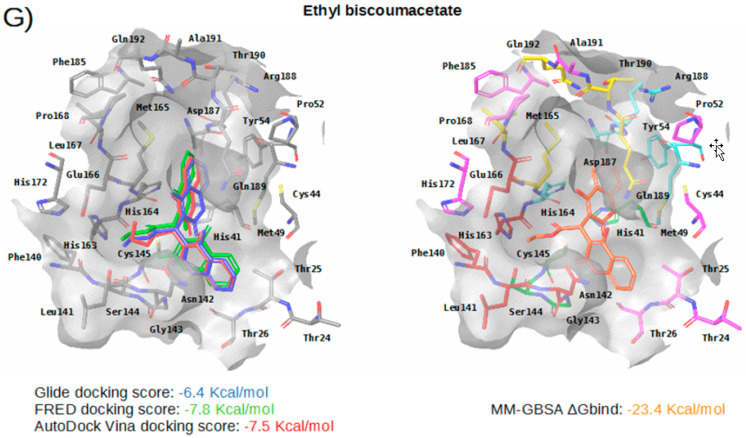
Panels (**A**–**G**) show results for Perampanel, Carprofen, Celecoxib, Alprazolam, Trovafloxacin, Sarafloxacin and Ethyl biscoumacetate; respectively. For each predicted M-pro inhibitor, the figure shows: (**A**) the superposition of the three equivalent docked poses (if more than one was found for the same hit, the one that presented the highest mean docking score was chosen); and (**B**) the corresponding Glide docked pose after the MM-GBSA minimization. Docking score values obtained with Glide, FRED and AutoDock Vina are shown in the same color as the equivalent pose obtained with the corresponding program. The ∆G_bind_ energy value obtained after the MM-GBSA minimization of the Glide pose is also shown for each predicted inhibitor. This figure was obtained with the help of the Maestro program [24].

**Figure 6:**
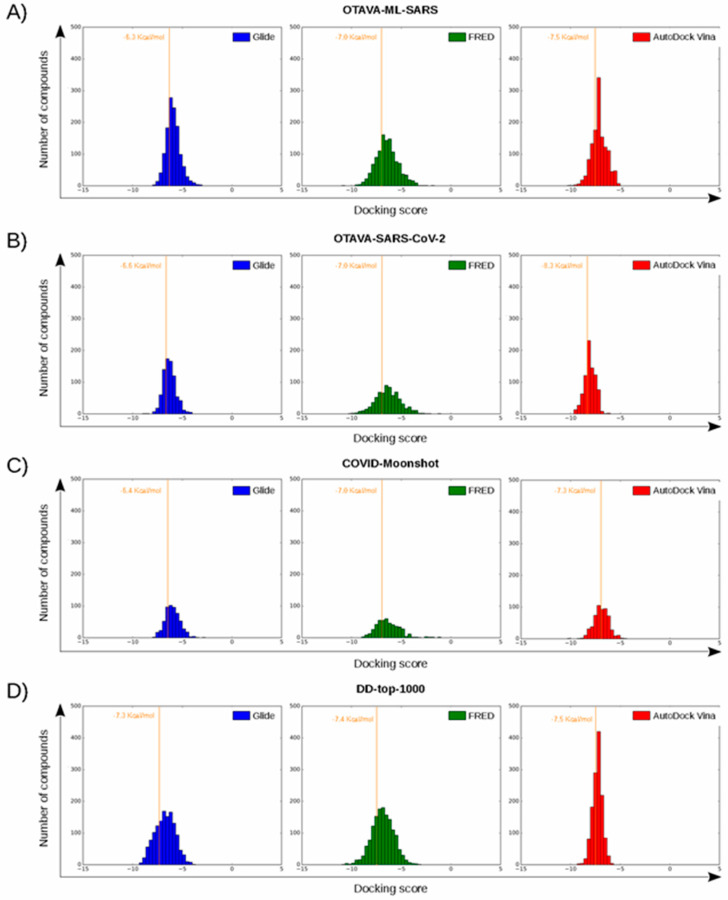
Histograms corresponding to the docking scores of four reference libraries containing predicted M-pro inhibitors. Panels (**A**–**D**) show the results for OTAVA-ML-SARS, OTAVA-SARS-CoV-2, COVID-Moonshot and DD-top-1000, respectively. Score thresholds for selecting the top 30% ligands with the highest affinity are shown for each histogram.

After applying the VS to two libraries of approved drugs (i.e., eDrug3D and Reaxys-marketed), seven potential M-pro inhibitors were identified: Perampanel, Carprofen, Celecoxib, Alprazolam, Trovafloxacin, Sarafloxacin and ethyl biscoumacetate (see Table 4). The three equivalent docked poses that presented the highest mean docking score for each hit compound and the result of the MM-GBSA minimization of the corresponding Glide pose at the M-pro binding site are shown in Figure 5. Interestingly, the docking scores obtained for our hit compounds were comparable to the four reference libraries of compounds designed specifically to inhibit M-pro (see Figure 5 and Figure 6). Although higher docking score values were obtained for many compounds in the DD-top-1000 library (see Figure 6D), the compounds at the eDrug3D and Reaxys-marketed libraries offer the advantage of having already been approved, and they should be ready to be used in a shorter period of time, which is crucial now that an urgent pharmacological treatment is needed for COVID-19. A description of the seven drugs that we predict as potential M-pro inhibitors and their predicted intermolecular interactions with M-pro is shown below.

Perampanel is an AMPA glutamate receptor antagonist used as an anticonvulsant to treat partial-onset seizures (see Table 4). Docking and energy minimization predict that Perampanel can interact with several residues of the S_3_, S_2_, S_1_, and S_1_’ subsites (see Table 5). In the predicted binding mode, its 2-pyridyl group effect hydrophobic interactions with Met165, Met49, Asp187 and His41 whereas the main chain oxygen of His164 establishes a hydrogen bond interaction with some hydrogen atoms from the 2-pyridyl ring (which should anchor the ligand to this pocket in a similar fashion to cocrystallized inhibitors; see Table 5 and Figure 5A). The 2-pyridyl group also effects a π stacking interaction with His41, further increasing the affinity of this substructure for this subpocket. Perampanel also establishes a hydrogen bond interaction with Gly143 close to the catalytic site, which is observed for all cocrystallized ligands (see Table 2). In a similar way to **13b**, **N3** and **X77**, Perampanel also establishes a hydrogen bond between hydrogens of one of its aromatic rings (i.e., the phenyl ring) and the main chain oxygen of Thr26. Finally, Leu141 uses its main chain oxygen in a hydrogen bond with the 2-cyanophenyl aromatic ring. This latter hydrogen bond is not present in the cocrystallized inhibitors (see Table 2) and, together with the one established by Thr26, helps to anchor Perampanel outside the hydrophobic pocket. Unfortunately, this drug presents severe side effects such as serious or life-threatening behavioral and psychiatric reactions. Considering these adverse effects, the risk-benefit ratio of this treatment option should be evaluated for COVID-19 patients at different stages of the disease after having confirmed its in vitro activity against M-pro.

Carprofen is a selective cyclooxygenase-2 (COX-2) inhibitor that was previously used as a pain reliever in the treatment of joint and postsurgical pain (see Table 4). In the binding site of M-pro, the minimized docked pose of Carprofen also occupies the hydrophobic pocket, effecting hydrophobic interactions with the residues Gln189, Met49, Asp187 and His41, and a hydrogen bond interaction with the main chain oxygen of His164. Moreover, its chloro group is able to establish a halogen bond interaction with the thiol group of the residue Cys44, which is present at the end of this subpocket, and a hydrophobic interaction with its side-chain CB atom (see Table 5 and Figure 5B). The ring system at the core of the compound is also predicted to effect a π-π interaction with His41. Carprofen also makes two additional hydrogen bonds with the main chain nitrogen atoms from Ser144 and Cys145 (while the former is not found in cocrystallized M-pro inhibitors, the latter has been described for **13a** and **13b**). According to the DrugBank database [36], this drug was withdrawn from the market in 1995 on commercial grounds. Carprofen was previously used in human medicine for over 10 years, and is approved for use in dogs. Regarding its adverse effects, Carprofen was generally well tolerated with mild adverse effects, i.e., similar to those of aspirin and other nonsteroidal anti-inflammatory drugs (NSAIDs). However, NSAIDs increase the risk of cardiotoxicity [37] and may worsen the course of community-acquired pneumonia [38]. Recent bioactivity data obtained for this compound by the COVID Moonshot initiative shows that Carprofen has limited inhibition capacity on SARS-CoV-2 M-pro (3.97% inhibition at 50 µM; see Table 4) and, therefore, could be used as a lead compound for the development of more potent inhibitors.

Celecoxib is a selective COX-2 inhibitor indicated for arthritis pain and to reduce precancerous polyps in the colon in familial adenomatous polyposis (see Table 4). The minimized docked pose of Celecoxib at the M-pro binding site places its hydrophobic trifluoromethyl group in the hydrophobic pocket, although only a hydrophobic interaction with Met49 is reflected in Table 5, possibly due to the smaller size of this substituent compared to those in the previous two compounds (see Figure 5C). Interestingly, the 4-sulfamoylphenyl group of this compound is predicted to occupy the S_1_ subsite and establish a hydrogen bond interaction with Asn142, which is also present in the cocrystallyzed complex structure with **X77**, as well as a hydrogen bond interaction with Leu141. Because of the anti-inflammatory actions of COX-2 inhibitors, the combination of an immunomodulatory agent, such as a thalidomide, and an anti-inflammatory agent, such as Celecoxib, has been suggested as a possible treatment of patients with severe COVID-19 pneumonia [39]. Although significant concerns regarding the safety of COX-2 selective NSAIDs emerged in the early 2000s, in 2005, the FDA concluded that the benefits of Celecoxib treatment outweighed the potential risks for properly selected and informed patients [40]. However, it is not advisable to administer Celecoxib or other NSAIDs to patients with previous cardiovascular events including acute myocardial infarction, coronary revascularization, or coronary stent insertion [41], and NSAIDs may worsen the course of community-acquired pneumonia [38]. Recent bioactivity data obtained for this compound by the COVID Moonshot initiative shows that Celecoxib has better inhibition capacity on SARS-CoV-2 M-pro than Carprofen (11.90% inhibition at 50µM; see Table 4) and it could also be used as a lead compound for the development of more potent inhibitors (for instance, by improving the capacity of its analogs to establish better interactions with the hydrophobic pocket at the S_3_ and the S_2_ subsites or with S_1_’).

Alprazolam acts on the receptors BNZ-1 and BNZ-2 and is used for the treatment of anxiety and panic disorders (see Table 4). In the minimized predicted binding mode of Alprazolam, its phenyl group is buried in the hydrophobic pocket, thus establishing hydrophobic interactions with Met165, Gln189, Met49, Asp187 and His41; additionally, it establishes an aromatic hydrogen bond with His164 (see Table 5 and Figure 5D). Moreover, the core of the molecule establishes a hydrogen bond interaction with the main chain nitrogen of Gly143 and with the main chain oxygen of Glu166 (see Table 5 and Figure 5D). All of these interactions have been observed in cocrystallized ligands (see Table 2) and should contribute to a good binding affinity. Moreover, the ring system of the phenyl moiety is also predicted to effect a π-π interaction with His41 (see Table 5 and Figure 5D). If the binding of Alprazolam to M-pro is confirmed, this drug could be advanced to clinical studies for COVID-19. According to the DrugBank database [36], Alprazolam is mainly metabolized by CYP3A and, thus, its administration together with CYP3A inhibitors like ketoconazole and itraconazole is contraindicated.

Trovafloxacin is a broad spectrum antibiotic that inhibits DNA gyrase and topoisomerase IV (see Table 4). In its predicted and minimized binding mode, the 2,4-difluorophenyl group of Trovafloxacin is buried in the hydrophobic pocket and effects hydrophobic interactions with Met165, Gln189 and Met49 (see Table 5 and Figure 5E). Interestingly, its carboxylic acid group is predicted to establish a hydrogen bond interaction with the main chain nitrogen of Ser144 and Cys145 (the latter also present in the complex with **13a** and **13b**; see Table 2). Additionally, the side chain of Gln189 is also involved in a hydrogen bond with the core of Trovafloxacin (see Table 5 and Figure 5E). However, according to the DrugBank database [36], this drug was withdrawn from the market due to its hepatotoxic potential, and therefore, it does not seem to be a relevant candidate for the treatment of COVID-19.

Sarafloxacin is a quinolone antibiotic (see Table 4). The predicted and minimized binding mode of Sarafloxacin is very similar to that of Trovafloxacin (see Table 5 and Figure 5E,F). The 4-fluorophenyl group of Sarafloxacin establishes hydrophobic interactions with the residues Met165, Gln189, Met49, Asp187 and His41 in the hydrophobic pocket, and it establishes an aromatic hydrogen bond with the main chain oxygen of His164 (see Table 5). The 4-fluorophenyl group also effects a π stacking interaction with His41, which should increase its affinity for this subpocket. The carboxylic acid group of Sarafloxacin is predicted to establish a hydrogen bond interaction with the side chain nitrogen from His41 and with the main chain nitrogen of Gly143 (the latter present in all cocrystallized M-pro complexes with inhibitors; see Table 2). Finally, Sarafloxacin is also hydrogen bonded to the main chain oxygen of Thr190 (a situation that is also found for **N3**; see Table 2 and Table 5). According to the DrugBank database [36], Sarafloxacin was discontinued by its manufacturer before receiving approval for its use in the US and Canada. Therefore, even if its M-pro inhibitory activity is confirmed, more data about its putative adverse effects would be necessary before being considered as a candidate for the treatment of COVID-19.

Ethyl biscoumacetate is a vitamin K antagonist used as an anticoagulant (see Table 4). In the predicted and minimized binding mode of ethyl biscoumacetate, one of its two 4-oxido-2-oxochromen-3-yl groups is oriented towards the hydrophobic pocket of the M-pro binding site, effecting hydrophobic interactions with Met165, Gln189, Met49 and His41, and hydrogen bond interactions with the His41 sidechain. In contrast, the other 4-oxido-2-oxochromen-3-yl group is oriented towards the S_1_’ pocket, and establishes a hydrogen bond interaction with the main chain nitrogen of Gly143 that is a common anchor point for cocrystallized M-pro inhibitors (see Table 2 and Table 5 and Figure 5G). Despite effecting similar interactions to the other VS hits and cocrystallized ligands, the structure of this compound is quite different from those of the other VS hits, as it does not present a central cyclic core; instead, it presents a central carbon atom that forks into three radical groups that occupy the S_3_, S_1_’ and S_1_ subsites. However, since according to the DrugBank database [36], ethyl biscoumacetate was withdrawn from the market and it can produce prolonged bleeding and severe hemorrhage, the risk-benefit ratio of this treatment option should be evaluated for COVID-19 patients at different stages of the disease after having confirmed its in vitro activity against M-pro.

To summarize, all the VS hits establish similar interactions in the binding site of M-pro. Firstly, most of them contain a hydrophobic substructure buried in the hydrophobic pocket of the enzyme present in the S_3_ and S_2_ subsites (with Celecoxib interacting only with the S_2_ subsite moiety of this hydrophobic pocket). Secondly, most of them interact with residues in the S_1_’ subsite, either by establishing hydrogen bond interactions with His41, Gly143, Ser144 or Cys145, or a combination of them and a π-π interaction with His41 in some cases (the exception is Celecoxib, which does not interact with the S_1_’ subsite). In addition, the different structures of the compounds allow different interactions to occur that are not present in crystallized complexes, such as a hydrogen bond interaction with the main chain oxygen from Leu141 in the case of Perampanel and Celecoxib or a halogen bond interaction with Cys44 in the case of Carprofen. Interestingly, 42.7% of the compounds from the DD-top-1000 library (that, according to the three docking scores used in the present work, show the highest overall affinity for M-pro; see Figure 6D) effect intermolecular interactions with Leu141. This could therefore explain why Celecoxib (which, as mentioned, lacks some of the characteristic interactions with M-pro found in cocrystallized inhibitors and in the remaining VS hits) is able to interact with the M-pro binding site. 

Independently of the possible adverse effects that may limit the fast use of some of these 7 drugs, the experimental validation of their activity against M-pro would be very useful for further research aimed at obtaining new drugs against COVID-19. For the moment, the predicted inhibition of M-pro has been experimentally proven for two out of the seven drugs (i.e., Carprofen and Celecoxib), and the remaining five have also been submitted to the COVID Moonshot initiative, in which it is expected that they will be selected for the next rounds of bioactivity testing (see Table 4 for their corresponding COVID Moonshot IDs). Overall, of the seven drugs predicted in our VS to inhibit M-pro, three of them (Perampanel, Celecoxib and Alprazolam) could be considered for COVID-19 clinical trials (provided that a significant inhibitory activity against M-pro was confirmed also for Perampanel and Alprazolam). However, a risk-benefit analysis of each drug must be carried out as, according to the MM-GBSA calculation of these three drugs, Perampanel displays a higher binding affinity for M-pro (ΔG_bind_ = −63.9 Kcal/mol) than Alprazolam (ΔG_bind_ = −57.6 Kcal/mol) or Celecoxib (ΔG_bind_ = −42.1 Kcal/mol), but shows the most severe adverse effects among these drugs. Although the M-pro inhibitory activity of Celecoxib is not very high, its risk–benefit ratio for COVID-19 patients is yet to be evaluated. Nevertheless, after the recent controversy on whether ibuprofen and other NSAIDs could worsen the effects of the SARS-CoV-2 virus, the last update of the WHO on the 18th of March of 2020 claimed that “based on currently available information, WHO does not recommend against the use of ibuprofen”.

### 2.4. Selectivity of This Virtual Screening Workflow

**Figure 7:**
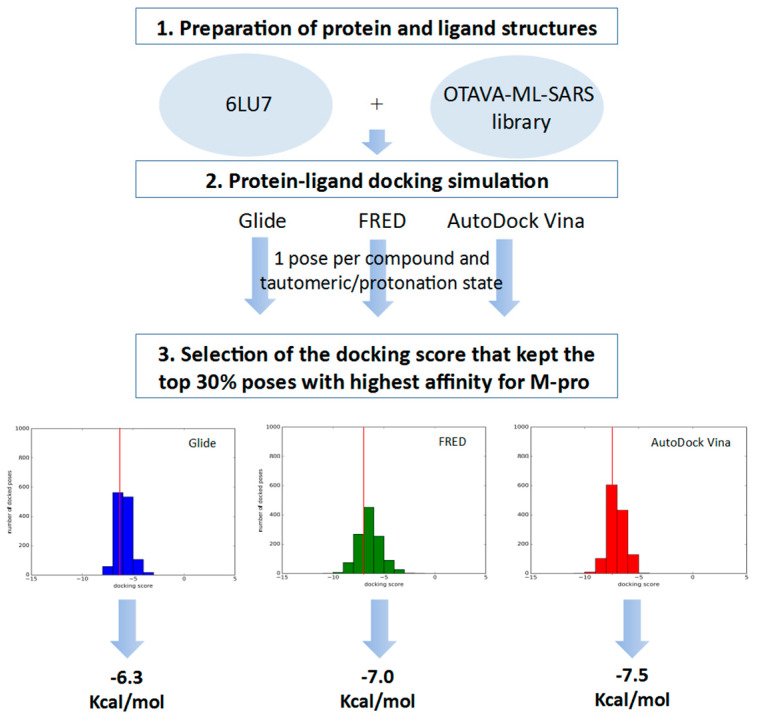
General scheme of the workflow used for defining the docking score thresholds to be used to identify high affinity poses for M-pro during the VS.

In order to check the validity of the bioactivity predictions made by this VS workflow, we checked how it performs on a sample comprising 28 experimentally known M-pro inhibitors (see Table S2) and 1600 calculated decoys. Figure S1 and Table S2 summarize the results obtained for each active in the VS. Two molecules could not be docked because of problems with either Omega (i.e., Disulfiram) or Glide (i.e., Ebselen). Among the remaining 26 M-pro inhibitors, only Shikonin had a triplet with all the comparisons between the three docked poses below the 1.5 Å threshold. Nevertheless, the docked poses involved in such triplet have more positive docking scores than the threshold values used for each protein-ligand docking program during the VS (see Table S2 and Figure 7). Therefore, none of the 26 M-pro inhibitors was recovered when using the same set up that was used for drug reposition.

Therefore, considering that the most reliable part of protein-ligand docking algorithms is their capacity to explore the hypothetical binding modes of a ligand at the binding site, we decided to identify the best triplet for each inhibitor and visually inspect the matching of the three poses without considering their docking scores. In Figure S1, we show how 8 out of 24 known M-pro inhibitors (i.e., 11a, Carmofur, Dipyridamole, Oxytetracycline, PX-12, Shikonin, Sulfacetamide and Tideglusib) have a triplet with equivalent poses and upper RSMD range values at the [1.29–2.64] Å interval. Interestingly, other M-pro inhibitors like Cimetidine, Maribavir or Omeprazole have similar values for the upper limit of the RMSD interval (i.e., 2.55, 2.32 and 2.56 Å, respectively), but the visual inspection of their corresponding triplets shows that the docked poses that form these triplets cannot be considered equivalent. Summarizing, a RMSD threshold around 2.5 Å could be used, but then a visual inspection of the triplets should be done in order to discard those that are not formed by equivalent poses.

Finally, when this 2.5 Å threshold was applied to the docked poses of the 1600 calculated decoys, triplets were found for only for 131 decoys. Considering that no visual inspection of these 131 triplets was done, this means that in these conditions (i.e., RMSD limited to 2.5 Å and no docking score threshold applied), the enrichment factor for the VS workflow is, at least, 3.6. Globally, this shows that during the reposition of the approved drugs, we used very strict conditions and, therefore, it is expected that the rate of false positives in that VS will be very low. This is in agreement with the fact that the only two VS hits in our study that have been experimentally tested (i.e., Celecoxib and Carprofen) are SARS-CoV-2 M-pro inhibitors. 

## 3. Materials and Methods

### 3.1. Libraries Description and Preparation

The following libraries of approved compounds were used for drug repositioning purposes: (a) e-Drug3D library: library of 1930 drugs and active metabolites approved by the FDA between 1939 and 2019 with a molecular weight ≤2000 Da from the database e-Drug3D [42]; and (b) Reaxys-marketed library: library of 4536 drugs labeled as “marketed” in the field “Highest clinical phase” from the Reaxys database [43].

The following libraries were used as a reference to establish key residue interactions and docking score cutoffs: (a) OTAVA-ML-SARS: library from OTAVA which contains 1577 compounds with predicted activity against SARS-CoV-2 based on machine learning approaches [44]; (b) OTAVA-SARS-CoV-2: library from OTAVA which contains 1017 compounds with predicted activity against SARS-CoV-2 M-pro based on receptor-based VS [44]; (c) COVID-Moonshot: library of 551 compounds mainly designed from cocrystallized drug fragments with SARS-CoV-2 M-pro and sent to COVID Moonshot before April 2020 [45]; and (d) DD-top-1000: a set of 1000 potential ligands for M-pro recently identified by applying Deep Docking to the ZINC15 database [46] and made publicly available by Ton et al. [5].

Compounds to be docked using Glide [47] and Autodock Vina [48] were prepared using the following instructions: (1) generate one 3D conformation per compound and discard the compounds with unspecified chiralities with Omega [49]; and (2) prepare the compounds for docking with LigPrep [50] by generating all the possible protonation states for each compound in the pH range 7.2 ± 1.0 and the default number of tautomers while respecting the chiralities from the input geometry of each compound. Compounds to be docked using Fred [51] were prepared using the following instructions: (1) set the ionization states of the compounds with *fixpka* [52]; (2) enumerate tautomeric forms with *tautomer* [52]; (3) assign atomic partial charges with *molcharge* [52]; and (4) generate one conformation per compound and discard compounds with unspecified chiralities with Omega [49].

### 3.2. Visual Inspection of the Fitting of Binding Site Coordinates to the Electron Density Maps

The correctness of the binding site coordinates was analyzed by visual inspection of how they fit to the corresponding EDM. This was done with the help of JMol 14.30.2 [53] and by using the EDMs (obtained from the PDBe at EMBL-EBI [54]) and their corresponding PDB files (obtained from the PDB database [54]). EDMs were displayed at 1.0 σ for all the M-pro binding site residues.

### 3.3. M-pro Structure Preparation, Grid Generation and Protein-ligand Docking Setup

The structure of M-pro in complex with the inhibitor **N3** was obtained from the Protein Data Bank (PDBid 6LU7). Before its preparation for each piece of docking software, the covalent bond with **N3** was removed. Preparation was performed with different tools, depending on the docking software used. During docking, only the best docked pose was generated for the reference libraries, whereas 10 poses were generated for each tautomer and protonation state of the compounds in the libraries of approved compounds used for drug repositioning purposes. In the case of dockings with Glide, the M-pro structure was prepared with Maestro [24] using Protein Preparation Wizard [55,56,57,58] with the following settings: (a) hydrogens were added after removal of original hydrogens; (b) N and C termini were capped; (c) disulfide bonds were created between sulfur atoms within 3.2 Å; (d) Epik [57] was used to generate probable tautomers and protonation states at a neutral pH; (e) H-bond assignment was further optimized using PROPKA [59] at a default pH value; (f) all water molecules were removed from the structure; and (g) the structure was minimized with the default force field. Glide was used to generate the grid around the cavity of the protein where the compounds were supposed to bind using the **N3** inhibitor bound to 6LU7 as a reference. The center coordinates corresponded to the centroid of **N3** (−10.36, 12.46, 68.7) and the box size was set to 35 x 35 x 35 Å. Glide was used to dock the different compound libraries to the M-pro structure by: (a) using standard-precision (SP) mode, and (b) generating 1 or 10 binding poses per compound depending on the library. In the case of dockings with FRED, MakeReceptor [51] was used to set up the receptor for docking by: (a) defining a box that enclosed the active site with its center coordinates and dimensions established based on the grid previously defined with Glide; (b) setting a shape potential; and (c) defining the inner and outer contours of the receptor with the default options. FRED 3.3.0.1. was used to dock the different compound libraries to the M-pro structure using the default settings to generate 1 or 10 binding poses per compound depending on the library. In the case of dockings with AutoDock Vina, AutoDockTools [60] from MGL Tools 1.5.6. was used to prepare the protein structure by: (a) removing all waters, and (b) adding polar hydrogens. The grid was defined as a box of the same size and center coordinates as in the other two docking programs and AutoDock Vina 1.1.2 was used to dock the different compound libraries to the M-pro structure using the default settings to generate 1 or 10 binding poses per compound depending on the library.

### 3.4. Identification of Equivalent Docked Poses among the Three Protein-Ligand Docking Programs

Equivalent docked poses among the three protein-ligand docking programs were identified by comparing the root mean square deviation (RMSD) between the heavy atoms of all docked poses obtained for the same tautomeric and protonation state of each molecule. If the RMSD between all possible pairs of docked poses in each triplet was less than or equal to 1.5 Å, the docked poses obtained with the three pieces of software were considered equivalent. The threshold value of 1.5 Å was chosen on the basis of visual inspection and general agreement in the literature [61]. The Schrödinger script *rmsd.py* [62] was used to calculate the RMSD values between poses.

### 3.5. Apply Docking Score Thresholds to Keep Only the Equivalent Docked Poses with the Highest Affinity for M-pro

The docking score thresholds chosen to select only the highest affinity equivalent poses were selected by docking the OTAVA-ML-SARS library with the same running conditions that were previously described for the approved compound libraries (with the only exception that for the OTAVA compounds only one docked pose for each tautomeric and protonation state was kept). Then, the docking score that kept only the top 30% of these compounds in each docking software (see Figure 7) was used as a threshold value to determine the minimum docking score that was regarded as indicating docked poses binding to M-pro with high affinity. These thresholds are −6.3, −7.0 and −7.5 Kcal/mol for Glide, FRED and AutoDock Vina, respectively (see Figure 6A and Figure 7).

Therefore, any of the equivalent docked poses of the approved compounds with docking scores more positive than their corresponding threshold were discarded (i.e., poses obtained with Glide, FRED or AutoDock Vina were compared, respectively with the Glide, FRED or AutoDock Vina thresholds). Then, only those approved compounds with a triplet of equivalent high affinity docked poses were considered VS hits (if more than one triplet of poses was found for the same hit, only the one that presented the highest mean docking score was chosen).

### 3.6. Virtual Screening Workflow Validation

The ability of the designed VS workflow to discern between active and nonactive molecules was evaluated by: (a) collecting from the literature all molecules with known in vitro activity as SARS-CoV-2 M-pro inhibitors (see Table S2); and (b) using this set of known M-pro inhibitors to obtain a set of 1600 decoys with the *Generate DUD•E Decoys* tool (http://dude.docking.org/generate). The docking of these two sets of molecules was performed with the same conditions that were previously described at Section 3.3 and 10 docked poses for each program were kept per tautomeric and protonation state.

### 3.7. Analysis of the Intermolecular Interactions between M-pro and Its Inhibitors

The Glide pose from each VS hit triplet was submitted to the MM-GBSA methodology available in Prime [58]. This methodology calculates the binding free energies (ΔG_bind_) from the predicted complexes obtained from ligand-docking simulations. During each MM-GBSA run, energy minimization was carried out to keep all protein residues frozen, except for a flexible region of 6 Å around the ligand. Otherwise, the remaining parameters used were the default values.

The coordinates of the protein-ligand complexes obtained after the MM-GBSA calculation were analyzed with the *poseviewer_interactions.py* script [24] to determine the intermolecular interactions between the docked poses and the M-pro binding site. The following interactions were analyzed: hydrogen bonds (HAccep, HDonor and Ar-Hbond), halogen bonds (XBond), salt-bridge interactions (Salt), π-cation interactions (PiCat), π-π interactions (PiFace, PiEdge) and hydrophobic interactions (HPhob). A similar approach, but without the use of MM-GBSA, was used with the top 30% ligands with the highest M-pro affinity from the four reference libraries containing predicted M-pro inhibitors. For the reversible and irreversible inhibitors cocrystallized with M-pro and available from the PDB database, the intermolecular interactions were also determined by applying the *poseviewer_interactions.py* script to the corresponding PDB files.

### 3.8. Analysis of Known Mutations of the M-pro Gene

A set of 2223 SARS-CoV-2 complete genomes was downloaded from GISAID [23]. These represented all the complete sequences with high coverage available on the 31st of March, 2020. The Wuhan-Hu-1/2019 genome (GenBank code MN908947.3) was used as a reference and for site numbering. A pairwise alignment between the M-pro gene, located between nucleotide positions 10,055 and 10,972 from the reference genome, and each of the complete genomes analyzed was obtained using the Smith-Waterman algorithm from BioPython. From these pairwise alignments, mutations from the reference genome were detected and classified as synonymous or missense mutations.

## 4. Conclusions

Finding drugs that can inhibit the infection caused by SARS-CoV-2 is an essential initial step while we wait for either herd immunity or a vaccine that can definitively stop this health emergency. Moreover, looking for such drugs among drugs that have already been approved is the fastest way to advance to clinical trials and spread their application among infected people. In this sense, computational approaches can make a great contribution by predicting which approved drugs have the best potential to be tested in vitro for COVID-19 repurposing. M-pro is an attractive target against SARS-CoV-2 because of its importance in the replication of SARS-CoV-2 virus, its conservation among other related viruses and a different cleavage specificity relative to human proteases [8].

In this manuscript, we developed a new VS procedure to predict novel M-pro inhibitors among approved drugs. This new approach consists of finding compounds that are predicted simultaneously by three docking programs (Glide, FRED and AutoDock Vina) to bind in the same manner and with high affinity to the active site of SARS-CoV-2 M-pro. This way, we took advantage of the conformational sampling algorithms of the three docking programs to generate hypothetic binding modes without relying on a single scoring function to rank the results. Interestingly, this is the first published VS on SARS-CoV-2 M-pro in which at least part of its in silico results were confirmed in vitro.

We predicted that seven drugs, i.e., Perampanel, Carprofen, Celecoxib, Alprazolam, Trovafloxacin, Sarafloxacin and ethyl biscoumacetate, can bind to the active site of SARS-CoV-2 M-pro in a similar way to known M-pro reversible and irreversible inhibitors and Carprofen and Celecoxib showed inhibition of M-pro in vitro. The drugs with few adverse effects, i.e., Perampanel, Celecoxib and Alprazolam, could be considered for COVID-19 clinical trials, provided that the inhibitory activity against M-pro for Perampanel and Alprazolam is also confirmed. However, a risk-benefit analysis of each drug must be conducted. Interestingly, due to its anti-inflammatory activity, Celecoxib in combination with thalidomide has also been suggested as a possible treatment of patients with severe COVID-19 pneumonia [39].

The analysis of the performance of known SARS-CoV-2 M-pro inhibitors and calculated decoys also confirms that we have applied a very strict criteria to perform the reposition of approved drugs. Therefore, it should be possible to repurpose more drugs (albeit at the risk of increasing the number of false positives) if the criteria are relaxed (i.e., using a higher RMSD threshold for the triplets and not considering the docking score threshold) and the RMSD calculation is complemented with a visual inspection of the resulting triplets. Moreover, the VS strategy we have developed could also be applied to commercial databases of nonapproved drugs to predict more putative SARS-CoV-2 M-pro inhibitors, thus increasing the number of available compounds for in vitro bioactivity assays against COVID-19. Finally, the analyses we show about interactions between M-pro and its inhibitors and the missense mutations found in the M-pro gene could be of interest for further research aimed at predicting new drugs for the treatment of COVID-19.

## Figures and Tables

**Table 1:** Synonymous and missense mutations of the M-pro gene from the analysis of 2223 complete genomes (high coverage only) available at GISAID [23] on 31st March, 2020.

Mutation	Type Mutation	AA Change
G10097A	missense	Gly15Ser
C10138T	synonymous	Asn28Asn
C10228T	synonymous	Leu58Leu
C10232T	missense	Arg60Cys
G10265A	missense	Gly71Ser
C10319T	missense	Leu89Phe
A10323G	missense	Lys90Arg
C10369T	synonymous	Arg105Arg
C10450T	synonymous	Pro132Pro
T10480C	synonymous	Asn142Asn
C10507T	synonymous	Asn151Asn
G10523A	missense	Val157Ile
C10572T	missense	Ala173Val
C10582T	synonymous	Asp176Asp
C10604T	missense	Pro184Ser
C10632T	missense	Ala193Val
C10641T	missense	Thr196Met
C10712T	missense	Leu220Phe
C10728T	missense	Thr225Ile
C10741T	synonymous	Asp229Asp
T10763C	missense	Tyr237His
T10771C	synonymous	Tyr239Tyr
C10789T	synonymous	Asp245Asp
C10818T	missense	Ala255Val
T10825A	synonymous	Thr257Thr
C10834T	synonymous	Ala260Ala
C10851T	missense	Ala266Val
G10870T	synonymous	Leu272Leu
A10874G	missense	Asn274Asp
A10912G	synonymous	Leu286Leu

**Table 2:** Summary of the intermolecular interactions in experimental complexes between M-pro and reversible and irreversible inhibitors. These interactions were obtained by applying the *poseviewer_interactions.py* script to the corresponding PDB files.

Subsite	Residue	13a 6Y7M	13b 6Y2F, 6Y2G	N3 6LU7	X77 6W63
S_3_	Met165	CB^h^, SD^h^	CB^h^, ^1^SD^h^	CB^h^,SD^h^	CB^h^
	Leu167				
	Gln189	CG^h^	CB^h^, CG^h^	CG^h^	CB^h^, CG^h^
	Thr190			O^d^	
	Gln192				
S_2_	Met49	CE^h^, SD^h^	CE^h^, ^2^SD^h^		CB^h^, CG^h^, SD^h^
	Tyr54				
	His164		O^d^	O^d^	O^Ar^
	Asp187	CB^h^	CB^h^		
	Arg188				
S_1_	Phe140	O^d^	O^d^	O^d^	
	Leu141				
	Asn142		CB^h^		OD1^Ar^
	His163	NE2^a^			NE2^a^
	Glu166	N^a^, O^d^	N^a^, O^d^, OE2^d^, CG^h^	N^a^, O^d^, OE2^s^	N^a^, CB^h^
S_1_’	His41	CB^h^	^1^NE2^d^, ^1^CB^h^	CB^h^, ^3^NE2^a^	CB^h^
	Gly143	N^a^	N^a^	N^a^	N^a^
	Ser144				
	Cys145	†SG, N^a^	†SG, N^a^	†SG, CB^h^	
	Thr25			CG2^h^	
	Thr26		O^Ar^	O^Ar^	O^Ar^
	Pro168		^1^CB^h^	CG^h^	
	His172		^3^CD2^a^	^3^CD2^a^	

^1^ only for 6Y2F; ^2^ only for 6Y2G. ^3^ An aromatic hydrogen bound to this atom is acting as hydrogen bond donor. Interactions are indicated with the protein atom that is involved, and make reference to the role played by the ligand in the intermolecular interaction with that protein atom: ^a^ HAccep, ^Ar^ Ar-Hbond, ^d^ HDonor, ^h^ HPhob and ^s^ Salt. † Interaction through a covalent bond.

**Table 3:** Summary of the intermolecular interactions between M-pro and the docked poses obtained by Glide of the top 30% ligands with the highest M-pro affinity from four reference libraries containing predicted M-pro inhibitors. Data shows the percentage of compounds for each library that is predicted to interact with each M-pro residue.

Sub-Site	Residue	OTAVA-ML-SARS	OTAVA-SARS-CoV-2	COVID-Moonshot	DD-top-1000
S3	Met165	88.5	91.7	67.1	66.4
	Leu167	4.2	5.8	11.4	5.9
	Gln189	95.5	92.1	90.4	96.4
	Thr190	9.7	14.5	15.0	11.2
	Gln192	2.9	3.7	7.8	1.3
S2	Met49	74.1	78.5	67.1	68.4
	Tyr54	0.3	0.0	0.6	4.3
	His164	17.3	18.2	49.1	76.3
	Asp187	25.9	22.3	38.3	47.3
	Arg188	14.1	14.0	10.2	30.5
S1	Phe140	12.3	7.4	14.4	18.8
	Leu141	14.4	22.7	14.4	42.7
	Asn142	18.3	19.8	22.8	9.2
	His163	4.5	3.3	4.8	4.8
	Glu166	50.0	62.0	70.7	59.8
S1′	His41	77.7	81.0	82.6	79.9
	Gly143	42.7	49.2	24.6	78.1
	Ser144	0.5	1.7	6.0	0.3
	Cys145	5.2	3.7	7.8	2.3
	Thr25	24.1	30.6	15.0	7.4
	Thr26	27.2	42.1	22.2	16.8
	Leu27	10.7	9.9	7.8	3.8
	Pro168	11.3	18.2	15.0	2.0

**Table 4:** Main characteristics of the seven drugs predicted as SARS-CoV-2 M-pro inhibitors.

Compound	Drugbank and COVID MoonShot IDs (with % of Inhibition at 50 µM When Available)	Status	Mechanism	Indication	Adverse Effects
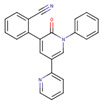 Perampanel	DB08883 GER-UNI-cfb	Approved	AMPA glutamate receptor antagonist.	Anticonvulsant: treatment of partial-onset seizures that may or may not occur with generalized seizures	Serious or life-threatening behavioral and psychiatric reactions
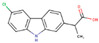 Carprofen	DB00821 GER-UNI-ec7-1 (3.97 ± 0.60%)	Approved; Withdrawn ^1^	selective cyclooxygenase-2 (COX-2) inhibitor	Pain reliever in the treatment of joint pain and postsurgical pain	Mild, such as gastro-intestinal pain and nausea, similar to those recorded with aspirin and other nonsteroidal anti-inflammatory drugs (NSAIDS)
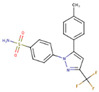 Celecoxib	DB00482 GER-UNI-05c (11.90 ± 0.59%)	Approved	selective COX-2 inhibitor	Arthritis pain and in familial adenomatous polyposis (FAP) to reduce precancerous polyps in the colon	Like other NSAIDS it is not advisable to administer it to patients with previous cardiovascular events
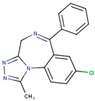 Alprazolam	DB00404 GER-UNI-cad	Approved	acts on benzodiazepine receptors BNZ-1 and BNZ-2	Treatment of anxiety and panic disorders	Generally related to its sedative effects. Mixed with alcohol it may lead to coma and death
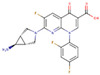 Trovafloxacin	DB00685 GER-UNI-c28	Approved; Withdrawn	inhibition of DNA gyrase and topoisomerase IV.	Broad spectrum antibiotic	It was withdrawn in 1999 due to its hepatotoxic potential.
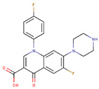 Sarafloxacin	DB11491 GER-UNI-cae	Vet approved; Withdrawn ^2^		Antibiotic	
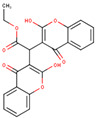 Ethyl biscoumacetate	DB08794 GER-UNI-9e0	Withdrawn	Vitamin K anatgonist	Anticoagulant	It is contraindicated in conditions like myocardial infarction, liver diseases, postpartum, hypersensitivity, pregnancy, bleeding, kidney disease, breast feeding and duodenal ulcer. It can produce increased blood clotting time, prolonged bleeding and severe hemorrhage.

Data were obtained from DrugBank (https://www.drugbank.ca/). ^1^ It is no longer marketed for human usage, after being withdrawn in 1995 on commercial grounds. ^2^ It was discontinued in 2001 by its manufacturer, Abbott Laboratories, before receiving approval for use in the US and Canada.

**Table 5:** Summary of the intermolecular interactions between M-pro and the seven compounds that we identified as putative M-pro inhibitors. These interactions were obtained with the *poseviewer_interactions.py* script after the Glide poses and the M-pro binding site were submitted to a MM-GBSA minimization.

Subsite	Residue	Perampanel	Carprofen	Celecoxib	Alprazolam	Trovafloxacin	Sarafloxacin	Ethyl Biscoumacetate
S_3_	Met165	CB^h^			CB^h^	CB^h^	CB^h^	CB^h^, SD^h^
	Leu167							
	Gln189		CG^h^		CG^h^	NE2^a^, CG^h^	CG^h^	CG^h^
	Thr190						O^d^	
	Gln192							
S_2_	Met49	CE^h^, SD^h^	CB^h^, CG^h^, SD^h^	SD^h^	CB^h^, CE^h^, CG^h^, SD^h^	CG^h^, SD^h^	CB^h^, CG^h^, SD^h^	SD^h^
	Tyr54							
	His164	O^Ar^	O^d^		O^Ar^		O^Ar^	
	Asp187	CB^h^	CB^h^		CB^h^		CB^h^	
	Arg188							
S_1_	Phe140							
	Leu141	O^Ar^		O^Ar^				
	Asn142			OD1^d^, CB^h^				CB^h^
	His163							
	Glu166	CB^h^		CB^h^	O^Ar^	CB^h^		
S_1_’	His41	CG^p^, CB^h^	CG^p^, CG^p^, CB^h^		CG^p^, CB^h^		NE2^a^, CB^h^, CG^p^	NE2^a^, CD2^Ar^, CB^h^
	Gly143	N^a^			N^a^		N^a^	N^a^
	Ser144		N^a^			N^a^		
	Cys145		N^a^			N^a^		
	Thr26	O^Ar^						
	Cys44		SG^x^, CB^h^					
	Pro52		CG^h^					

Interactions are indicated with the protein atom that is involved and make reference to the role played by the ligand in the intermolecular interaction with that protein atom: ^a^ HAccep, ^Ar^ Ar-Hbond, ^d^ HDonor, ^h^ HPhob, ^p^ PiFace and ^x^ XBond.

## Supplementary Materials

Supplementary materials can be found at https://www.mdpi.com/1422-0067/21/11/3793/s1 and consist on two tables and one figure. Table S1 summarizes the PDB codes for SARS-CoV-2 M-pro (updated on 30.03.20). Table S2 summarizes which compounds have been experimentally identified as M-pro inhibitors and reports their corresponding: (a) IC_50_ value; (b) inhibition index in infected cells; (c) docking scores for the best triplet; and (d) RMSD range for all the comparisons within the triplet. Figure S1 shows the 2D structure of the M-pro inhibitors reported at Table S2 and the superposition for the best triplet obtained for all of them (see the corresponding docking score values and their RMSD range values also at Table S2).
