# Supplementary material for: Prediction of Novel Inhibitors of the Main Protease (M-pro) of SARS-CoV-2 through Consensus Docking and Drug Reposition
Source: Int J Mol Sci. 2020 May 27;21(11):3793. doi: 10.3390/ijms21113793 (PMC7312484; doi:10.3390/ijms21113793)
Supplement: Supplementary file 1 [file ijms-21-03793-s001.pdf]

Supplementary materials for

Prediction of novel inhibitors of the main protease (M-pro) of SARS-CoV-2 through consensus docking and drug reposition

Aleix Gimeno[1,‡], Júlia Mestres-Truyol[1,‡], María José Ojeda-Montes[2], Guillem Macip[1], Bryan Saldivar-Espinoza[1], Adrià Cereto-Massagué[1], Gerard Pujadas[1,3,*] and Santiago Garcia-Vallvé[1,3,*]

[1] Research group in Cheminformatics & Nutrition, Departament de Bioquímica i Biotecnologia, Universitat Rovira i Virgili, Campus de Sescelades, 43007 Tarragona, Catalonia, Spain
[2] Escoles Universitàries Gimbernat i Tomàs Cerdà, 08174 Sant Cugat del Vallès, Barcelona, Catalonia, Spain
[3] EURECAT, TECNIO, CEICS, Avinguda Universitat 1, 43204 Reus Catalonia (Spain)

‡ Both authors contributed equally to this study

* Correspondence: gerard.pujadas@gmail.com; santi.garcia-vallve@urv.cat

Table S1. Summary of PDB codes for SARS-CoV-2 M-pro (updated on 30.03.20).

Total structures: 87

Free-form (4)
6M03, 6Y2E, 6Y84, 6YB7

M-pro/inhibitor complex (63)

Covalently bound to M-pro through Cys145 (43)

Covalent inhibitor (4)
6LU7, 6Y2F, 6Y2G, 6Y7M

Covalent fragment (39)
5REJ, 5REK, 5REL, 5REM, 5REN, 5REO, 5REP, 5RER, 5RES, 5RET, 5REU, 5REV, 5REW, 5REX, 5REY, 5RFF, 5RFG, 5RFH, 5RFI, 5RFJ, 5RFK, 5RFL, 5RFM, 5RFN, 5RFO, 5RFP, 5RFQ, 5RFR, 5RFS, 5RFT, 5RFU, 5RFV, 5RFW, 5RFX, 5RFY, 5RFZ, 5RG0, 5RG2, 5RG3

Non-covalently bound to M-pro (20)

Non-covalent inhibitor (1)
6W63

Non-covalent fragment (19)
5R7Y, 5R7Z, 5R80, 5R81, 5R82, 5R83, 5R84, 5RE4, 5RE9, 5REB, 5REH, 5REZ, 5RF1, 5RF2, 5RF3, 5RF6, 5RF7, 5RFE, 5RG1

Other interactions (20)

Fragment in the dimer interface (2)
5RF0, 5RFA

Fragment on the surface (3)
5REC, 5REE, 5RF5

Fragment in crystal contacts(15)
5RED, 5RE5, 5RE6, 5RE7, 5RE8, 5REA, 5REF, 5REG, 5REI, 5RF4, 5RF8, 5RF9, 5RFB, 5RFC, 5RFD

Table S2. Summary of *in vitro* data for known SARS-CoV-2 M-pro inhibitors (updated on 10.05.20).

Drug name	IC50 (μM)	Inhibition index [5]	Glide score (kcal/mol)	FRED score (kcal/mol)	AutoDock Vina score (kcal/mol)	RMSD range (Å)
**11a**	0.05 ± 0.005 [3]		-8.16	-13.05	-7.50	[0.64-2.10]
**11b**	0.04 ± 0.002 [3]		-6.26	-10.68	-8.80	[0.93-4.56]
**11r**	0.18 ± 0.02 [4]		-7.48	-12.65	-6.50	[1.68-4.40]
**13a**	2.39 ± 0.63 [4]		-5.30	-0.52	-7.70	[2.10-5.30]
**13b**	0.67 ± 0.18 [4]		-5.23	-0.35	-8.40	[3.06-4.99]
**Atazanavir**	7.53 ± 0.31 [1]		-4.28	1.31	-6.00	[4.40-9.42]
**Candesartan**	9.45 ± 0.73 [1]	1.166	-4.02	-3.25	-7.70	[1.74-4.35]
**Candesartan Cilexetil**	2.78 ± 0.31 [1]		-6.09	-3.61	-7.00	[5.47-6.53]
**Carmofur**	1.82 ± 0.06 [2]	0.054	-4.99	-4.71	-6.40	[0.99-2.64]
**Chloroquine**	7.16 ± 0.23 [1]	1.351	-4.51	-6.62	-5.40	[2.69-4.43]
**Cimetidine**	~ 50 [1]	0.014	-1.87	-4.26	-5.00	[1.27-2.55]
**Cinanserin**	124.93 ± 7.89 [2]		-6.15	-7.94	-6.10	[2.16-4.53]
**Dipyridamole**	0.55 ± 0.01 [1]	0.086	-4.60	-4.36	-6.60	[1.50-2.24]
**Disulfiram**	9.35 ± 0.18 [2]<br>4.67 ± 0.42 [1]	-0.116	N/A	N/A	N/A	N/A
**Ebselen**	0.67 ± 0.09 [2]	0.271	N/A	N/A	N/A	N/A
**Indinavir**	43.13 ± 2.79 [1]	0.382	-6.62	1.69	-8.00	[4.46-5.54}
**Maribavir**	~ 50 [1]		-6.21	-5.87	-8.10	[1.20-2.32]
**Montelukast**	7.29 ± 0.54 [1]	0.339	-5.29	-2.76	-8.00	[3.06-7.17]
**N3**	(a) [2]		-6.58	3.16	-8.50	[4.47-7.82]

**Omeprazole**	20.96 ± 0.95 [1]	1.249	-6.18	-4.75	-6.20	[1.38-2.56]
**Oxytetracycline**	15.15 ± 0.85 [1]	0.403	-5.06	-3.16	-6.20	[1.35-2.21]
**PX-12**	21.39 ± 7.06 [2]		-4.58	-5.02	-4.20	[0.91-2.49]
**Roxatidine acetate**	20.31 ± 0.37 [1]	0.109	-4.20	-3.90	-5.70	[3.98-6.36]
**Shikonin**	15.75 ± 8.22 [2]		-5.11	-5.62	-6.50	[0.91-1.29]
**Sulfacetamide**	~ 50 [1]	0.002	-3.78	-4.51	-5.10	[1.37-1.63]
**Tideglusib**	1.55 ± 0.30 [2]		-5.75	-6.00	-6.70	[1.21-1.99]
**Ubenimex**	8.93 ± 1.21 [1]	0.012	-4.83	-5.63	-5.60	[2.08-3.24]
**Valganciclovir**	16.66 ± 0.87 [1]	0.237	-5.42	-4.91	-6.50	[3.29-4.04]

(a) $k_{obs}/[I]=11.300M^{-1}s^{-1}$

1. Li Z, Li X, Huang Y-Y, *et al.* FEP-based screening prompts drug repositioning against COVID-19. *bioRxiv*, 2020.03.23.004580 (2020). Available from: https://www.biorxiv.org/content/10.1101/2020.03.23.004580v1?ct=.

2. Jin Z, Du X, Xu Y, *et al.* Structure of Mpro from COVID-19 virus and discovery of its inhibitors. *Nature*. 368(6489), 409–412 (2020). Available from: https://www.sciencemag.org/lookup/doi/10.1126/science.abb3405.

3. Dai W, Zhang B, Su H, *et al.* Structure-based design of antiviral drug candidates targeting the SARS-CoV-2 main protease. *Science*. 4489(April), eabb4489 (2020). Available from: http://www.ncbi.nlm.nih.gov/pubmed/32321856.

4. Zhang L, Lin D, Sun X, *et al.* Crystal structure of SARS-CoV-2 main protease provides a basis for design of improved α-ketoamide inhibitors. *Science (80-.).* 3405(March), eabb3405 (2020). Available from: http://www.ncbi.nlm.nih.gov/pubmed/32198291.

5. Touret F, Gilles M, Barral K, *et al.* In vitro screening of a FDA approved chemical library reveals potential inhibitors of SARS-CoV-2 replication. *bioRxiv*. , 2020.04.03.023846 (2020). Available from: https://www.biorxiv.org/content/10.1101/2020.04.03.023846v1.

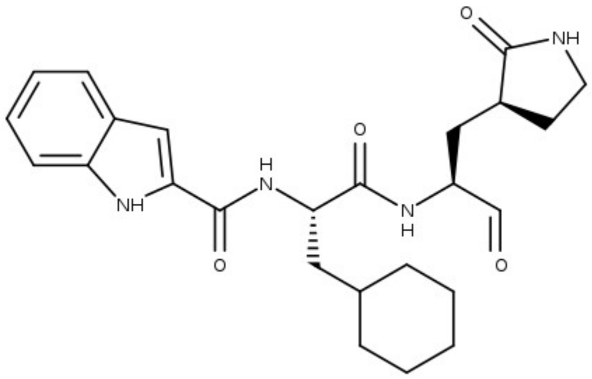

11a

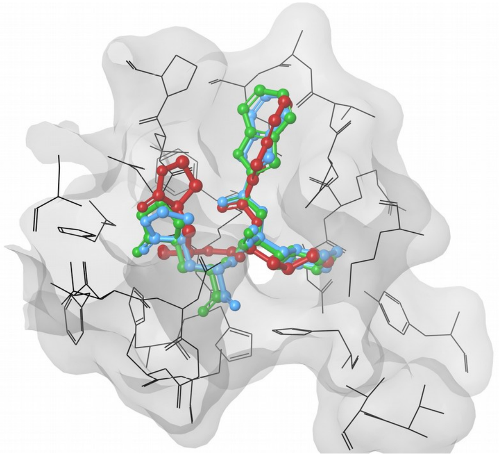

RMSD: 0.64 to 2.10

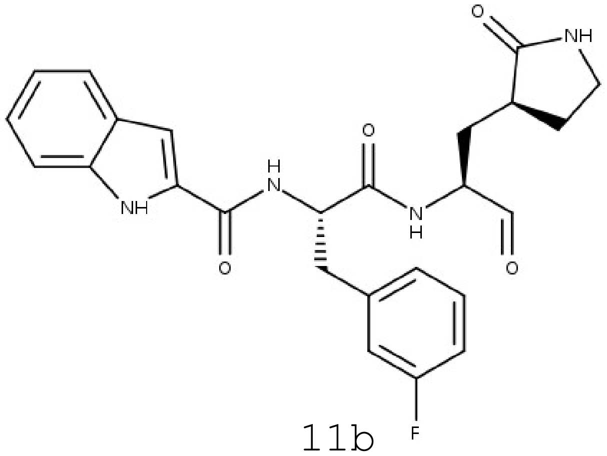

11b

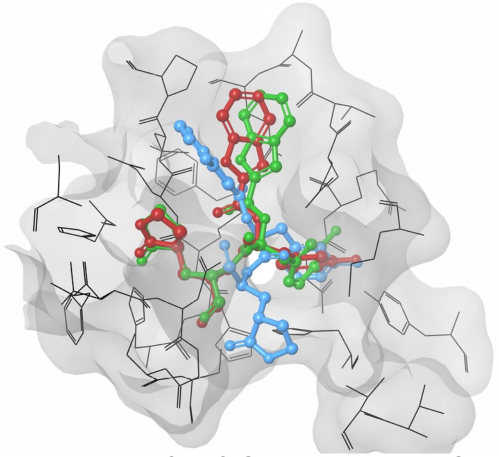

RMSD: 0.93 to 4.56

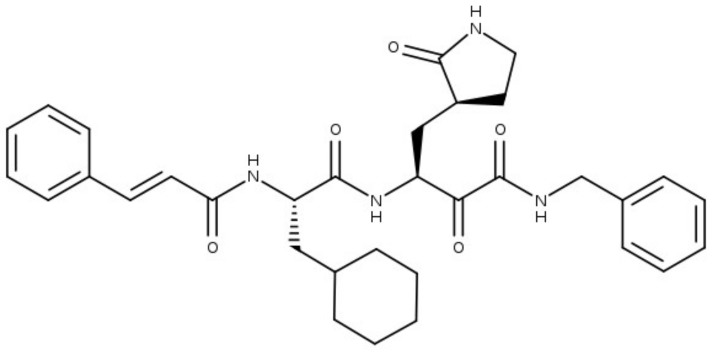

11r

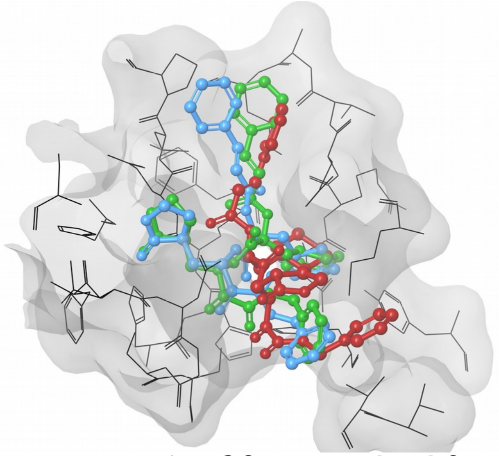

RMSD: 1.68 to 4.40

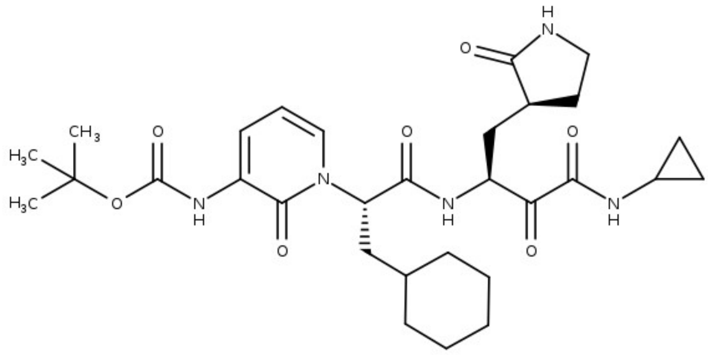

13a

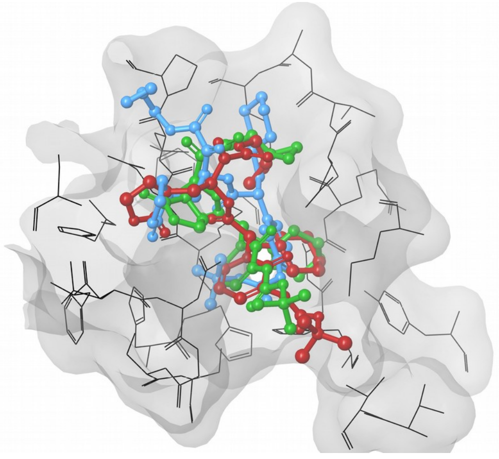

RMSD: 2.10 to 5.30

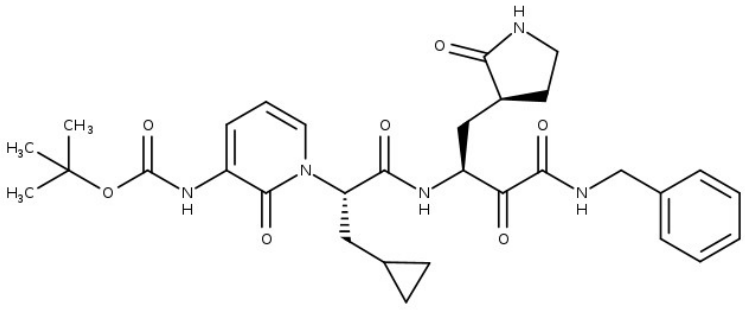

13b

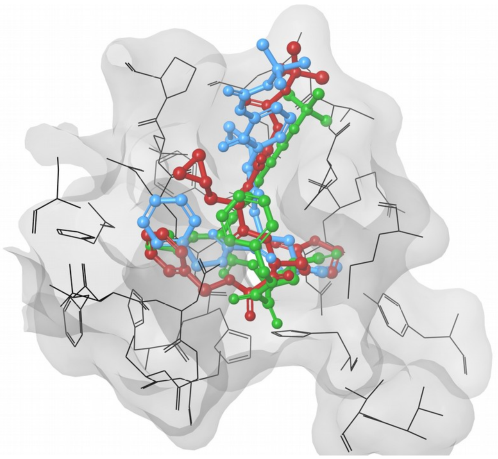

RMSD: 3.06 to 4.99

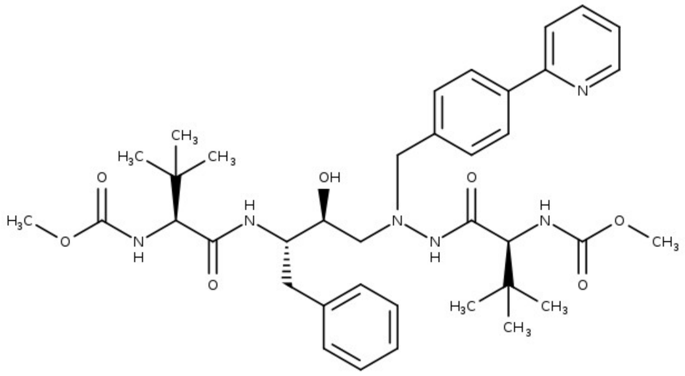

Atazanavir

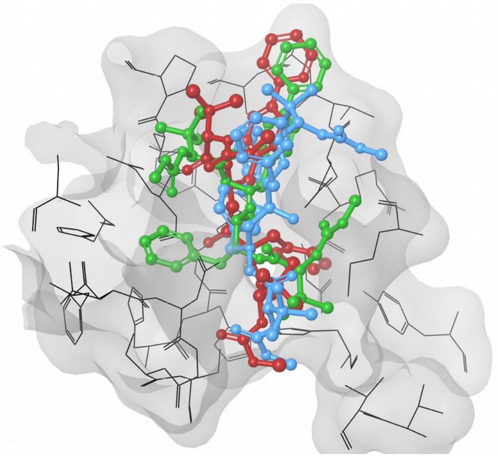

RMSD: 4.40 to 9.42

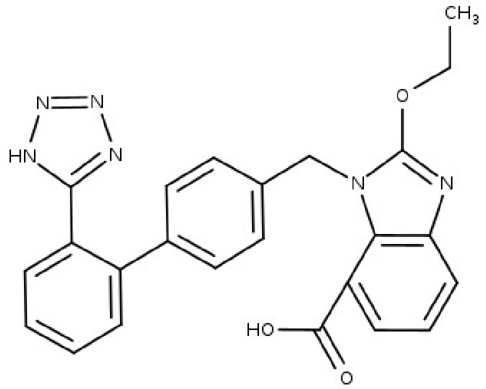

Candesartan

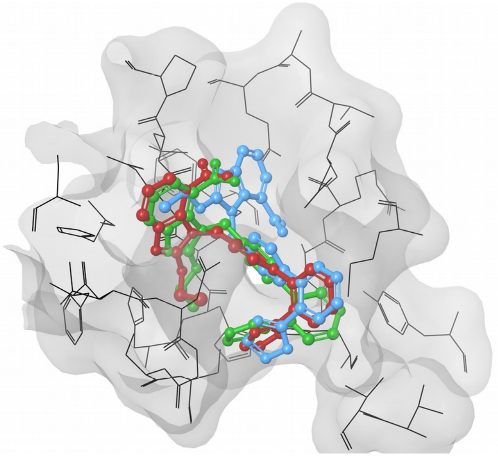

RMSD: 1.74 to 4.35

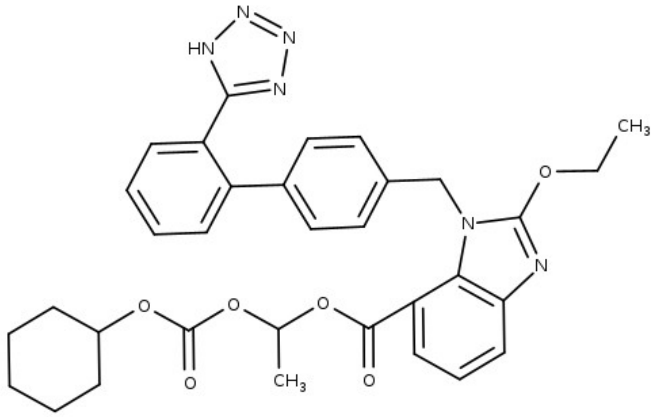

Candesartan Cilexetil

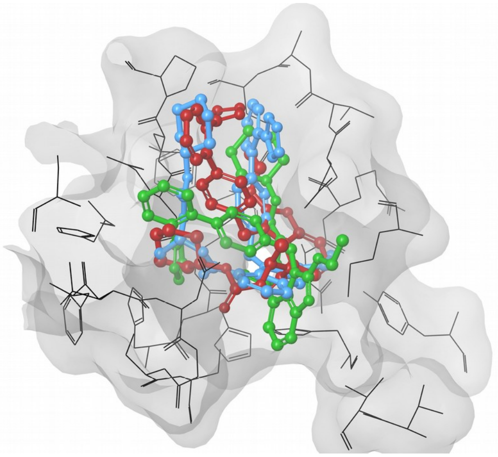

RMSD: 5.47 to 6.53

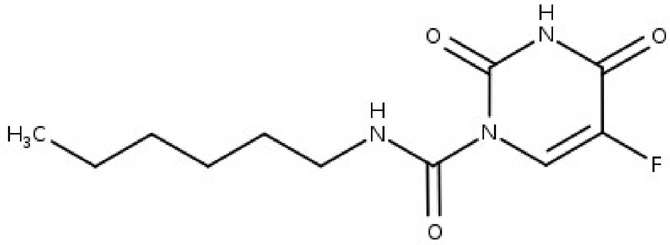

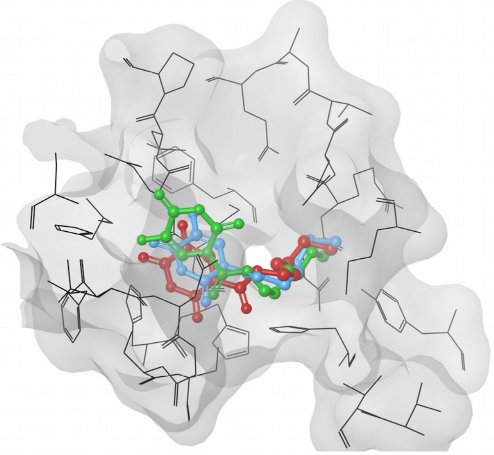

Carmofur

RMSD: 0.99 to 2.64

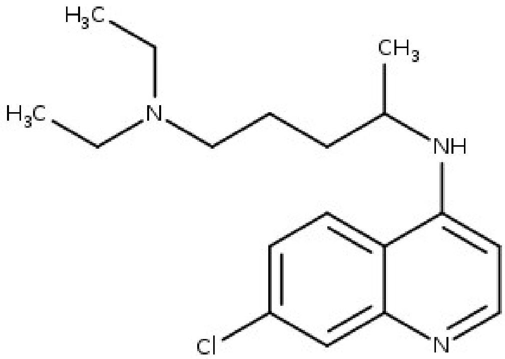

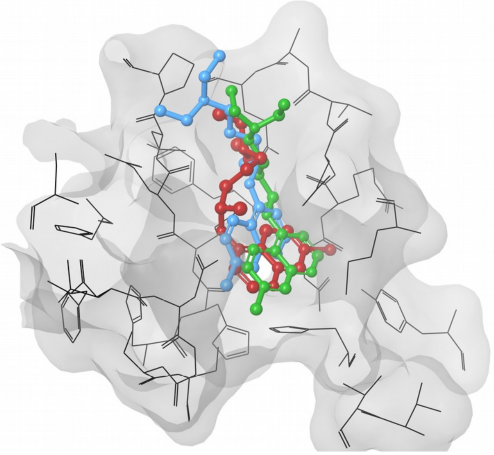

Chloroquine

RMSD: 2.69 to 4.43

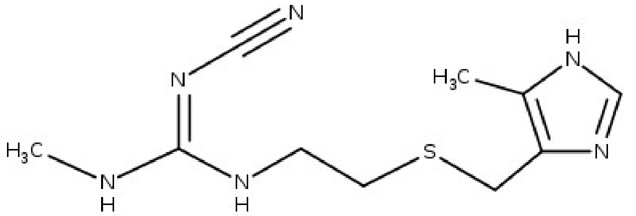

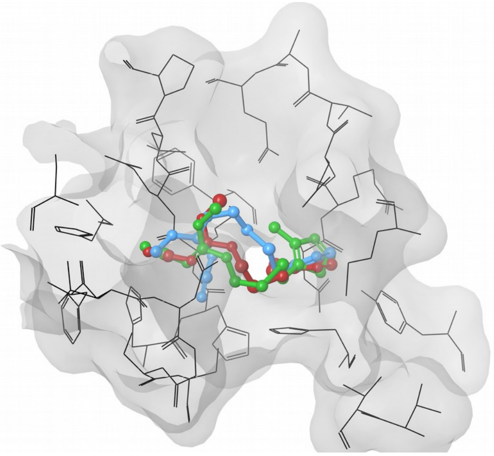

Cimetidine

RMSD: 1.27 to 2.55

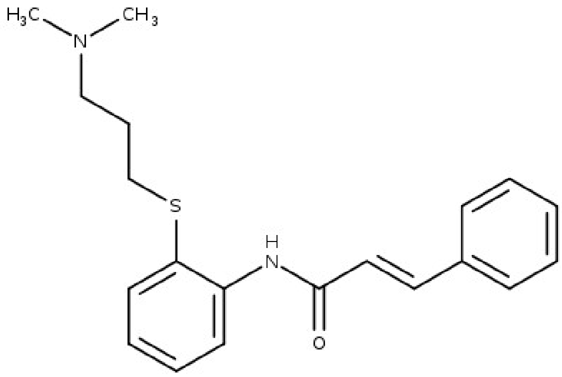

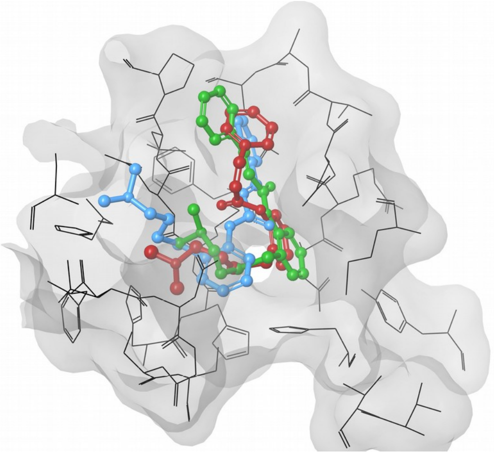

Cinanserin

RMSD: 2.16 to 4.53

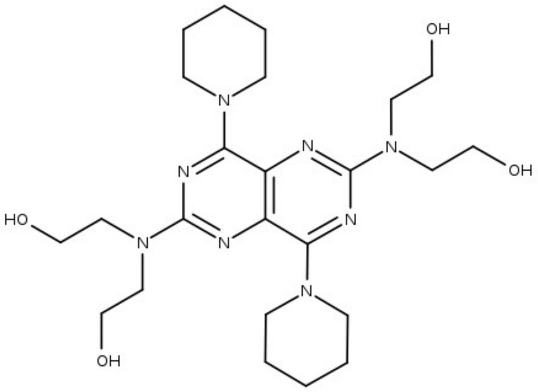

Dipyridamole

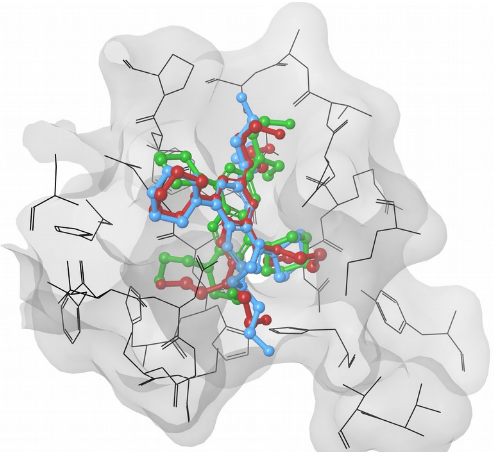

RMSD: 1.50 to 2.24

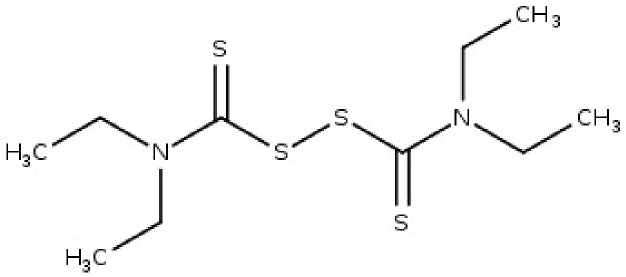

Disulfiram

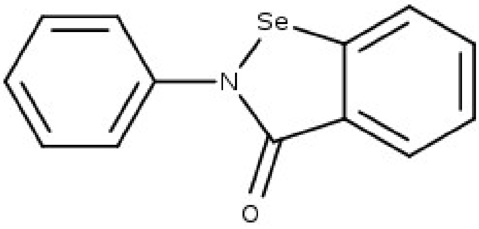

Ebselen

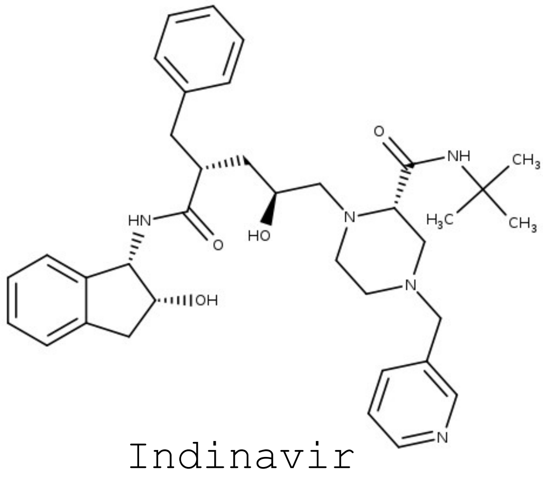

Indinavir

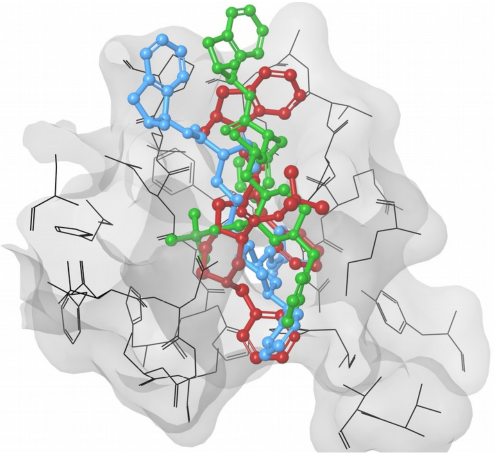

RMSD: 4.46 to 5.54

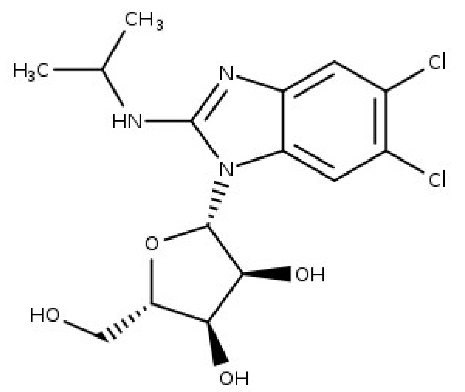

Maribavir

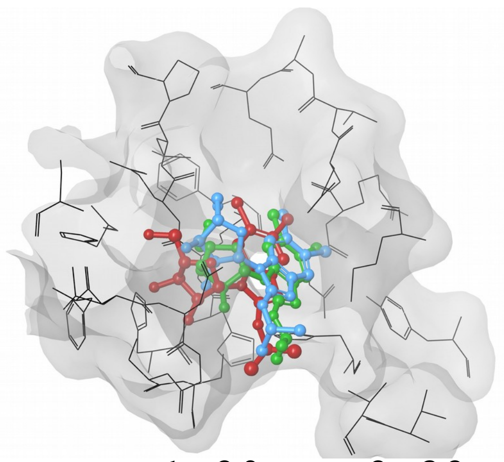

RMSD: 1.20 to 2.32

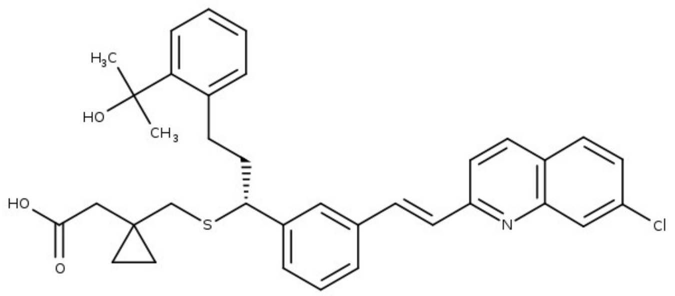

Montelukast

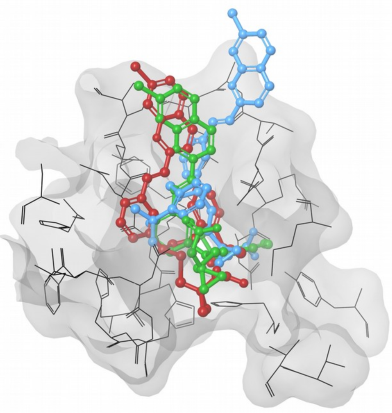

RMSD: 3.06 to 7.17

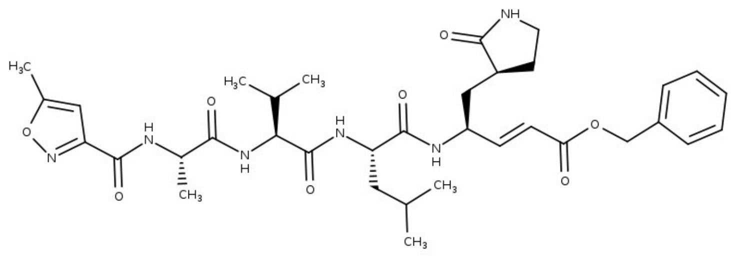

N3

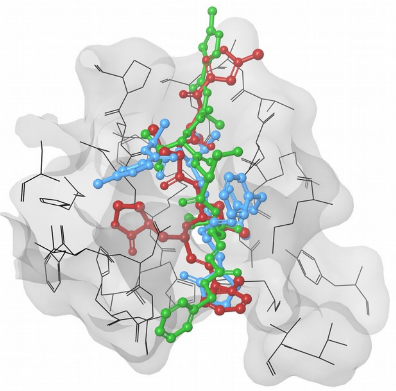

RMSD: 4.47 to 7.82

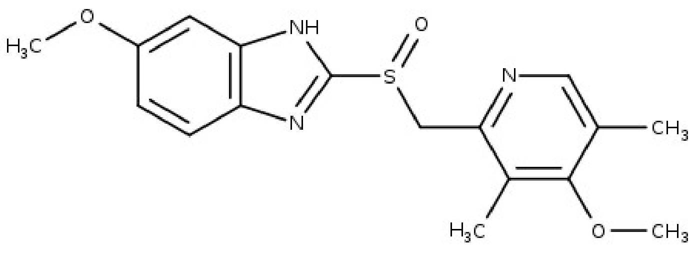

Omeprazole

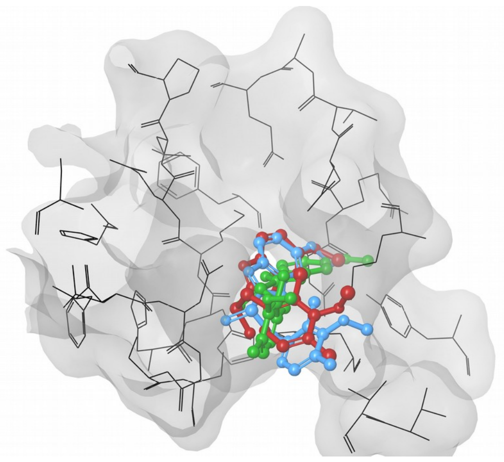

RMSD: 1.38 to 2.56

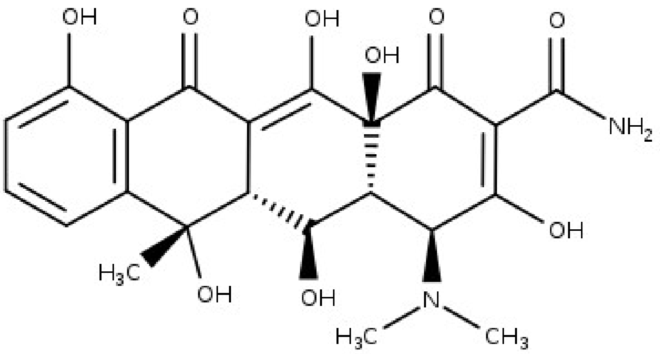

Oxytetracycline

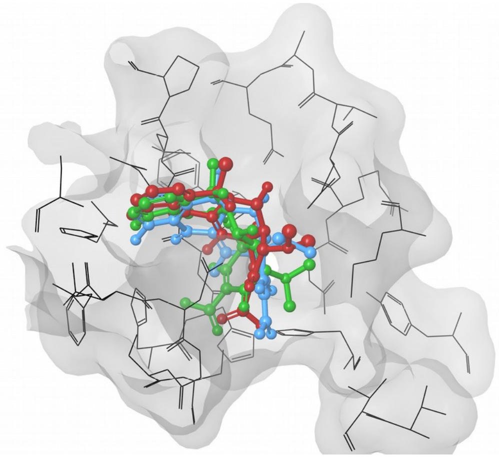

RMSD: 1.35 to 2.21

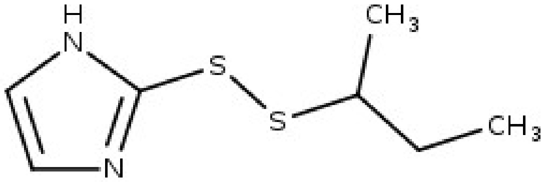

PX-12

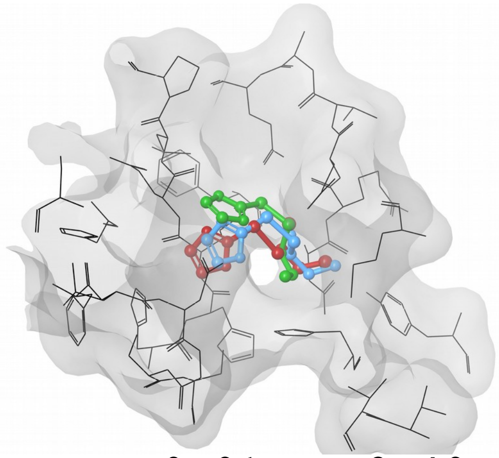

RMSD: 0.91 to 2.49

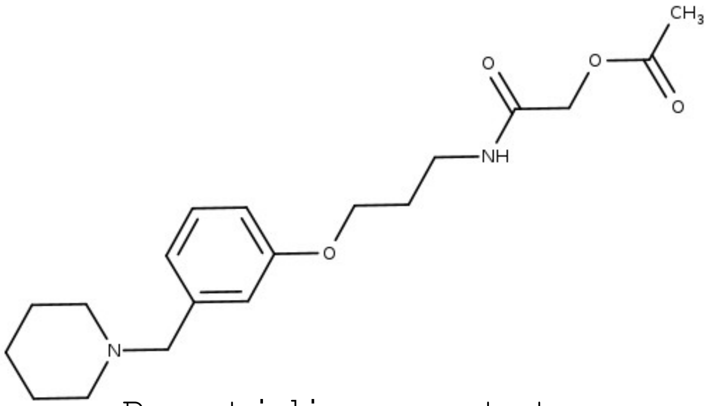

Roxatidine acetate

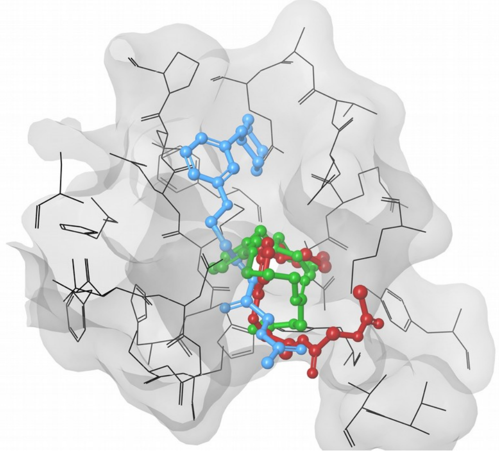

RMSD: 3.98 to 6.36

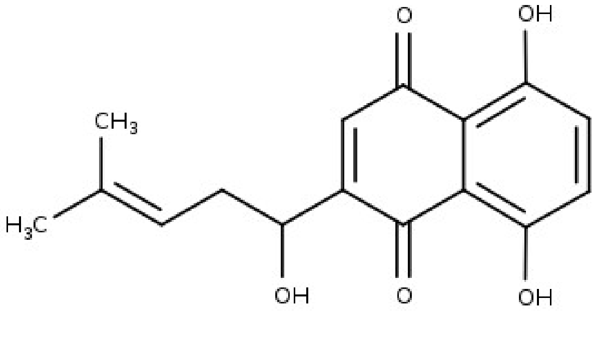

Shikonin

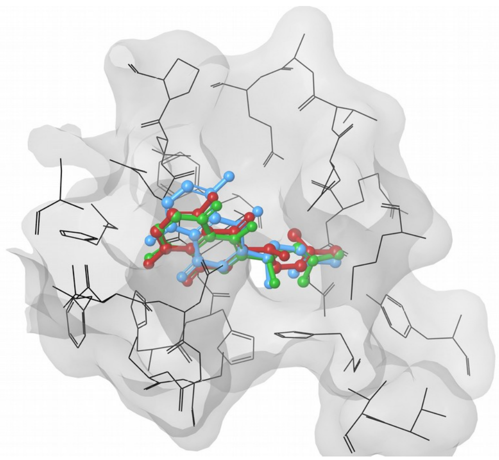

RMSD: 0.91 to 1.29

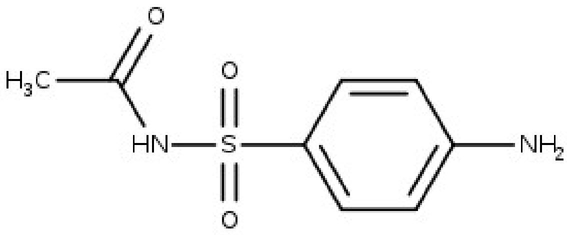

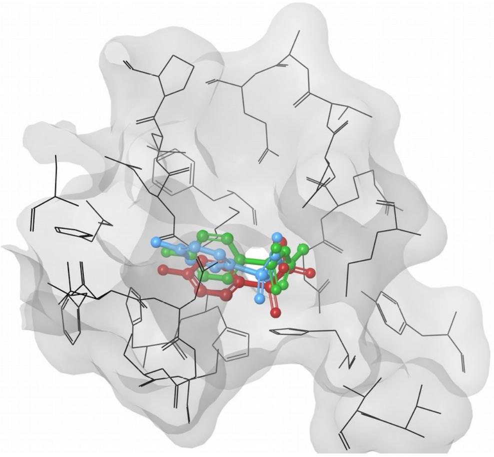

Sulfacetamide

RMSD: 1.37 to 1.63

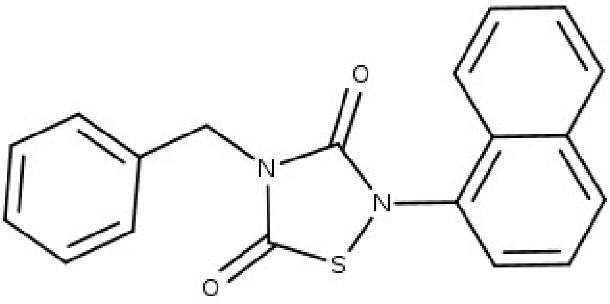

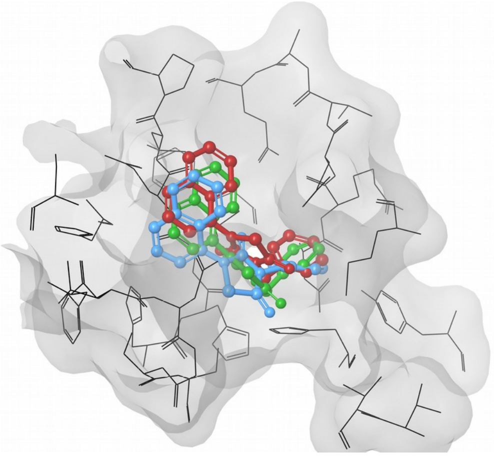

Tideglusib

RMSD: 1.21 to 1.99

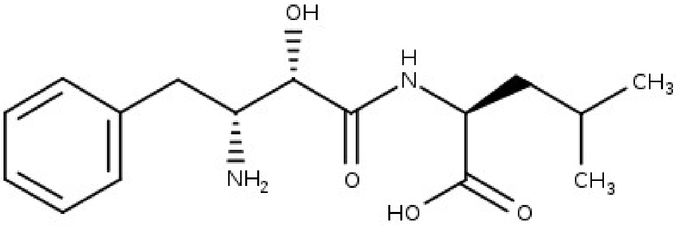

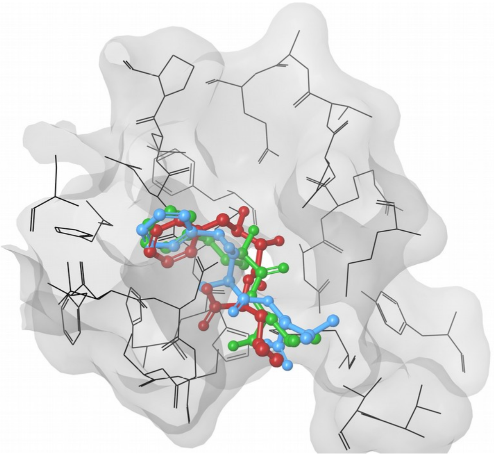

Ubenimex

RMSD: 2.08 to 3.24

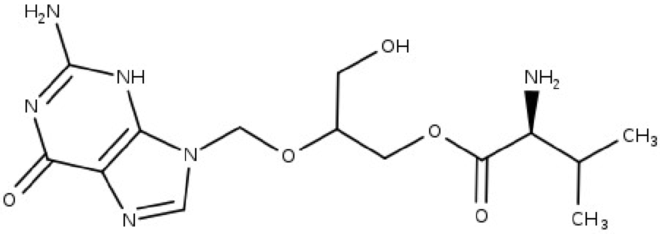

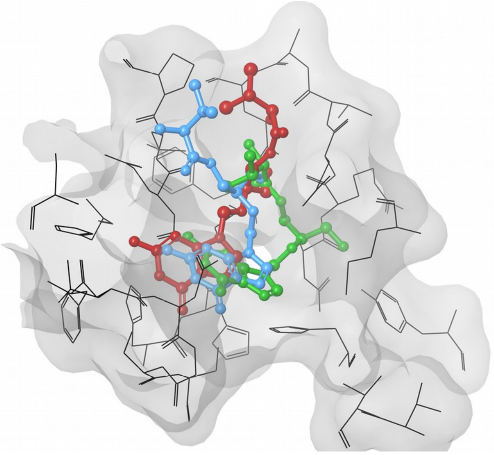

Valganciclovir

RMSD: 3.29 to 4.04

Figure S1. Each panel shows the 2D structure and the lowest-RMSD triplet for each known M-pro inhibitor in Table S2.
